# Inducible T-bet deletion reveals tissue-specific requirements in NKp46+ ILC immunobiology and response to murine cytomegalovirus

**DOI:** 10.1038/s41467-026-74478-y

**Published:** 2026-06-18

**Authors:** Pamela Wong, Yeeun Paik, Lily Chang, Allison Burdi, Carly Neal, Celia C. Cubitt, Jennifer Tran, Bishan Bhattarai, Lyra Morina, Nancy D. Marin, Kimberly Hwang, Hyogon Sohn, Julia A. Wagner, Jennifer A. Foltz, Wilbur M. Song, Timothy Schappe, Lynne Marsala, Mark Foster, Julia Foster, Melissa M. Berrien-Elliott, Sytse J. Piersma, Marco Colonna, Wayne M. Yokoyama, Megan A. Cooper, Todd A. Fehniger

**Affiliations:** 1 Division of Oncology, Department of Medicine, Washington University School of Medicine, St. Louis, MO USA; 2 Department of Pathology and Immunology, Washington University School of Medicine, St. Louis, MO USA; 3 Division of Rheumatology and Immunology, Department of Pediatrics, Washington University School of Medicine, St. Louis, MO USA; 4 Division of Rheumatology, Department of Medicine, Washington University School of Medicine, St. Louis, MO USA

**Keywords:** Innate lymphoid cells, Innate immunity, Viral infection

## Abstract

The requirement for the T-box transcription factors (TF) T-bet and Eomes in innate lymphoid cells (ILCs) beyond their development is not well understood. Here, we generate an inducible, NKp46-specific T-bet knock-out (KO) model and compare it to corresponding Eomes KO and combined T-bet Eomes double KO mice to define T-box TFs requirement in the homeostasis and function of mature NK cell and other NKp46^+^ ILC. Inducible T-bet deletion reduces stage IV NK cell numbers in the spleen and tissues, while preserving NK cells in the bone marrow and lymph nodes. Liver ILC1 and small intestine lamina propria NKp46^+^ ILC3 are also lost upon T-bet deletion, indicating the requirement for continuous T-bet expression in these ILC types. Combined T-bet and Eomes KO leads to a rapid loss of NK cells, markedly greater than with individual T-bet or Eomes KO. Direct comparison of inducible T-bet and Eomes KO models reveals that Eomes is critical for host protection against murine cytomegalovirus, whereas T-bet is dispensable, despite loss of ILC1. These findings establish the tissue-specific and non-redundant roles for T-box TFs in NKp46^+^ ILCs homeostasis and response to viral infection.

## Abstract (web summary)

The role of T-box transcription factors in NK cells and NKp46^+^ ILCs after their development is not well understood. Here, inducible knock-out of T-bet in mature NK cells and NKp46^+^ ILCs show tissue-specific requirements for T-bet in their maintenance and function. The authors further show that Eomes, but not T-bet, is required for MCMV protection.

## Introduction

NK cells are innate lymphoid cells (ILCs) that are important for host defense against infection and mediate anti-tumor responses^1,2^. Once activated by target cells or cytokines, NK cells proliferate, secrete cytokines and chemokines, and kill diseased target cells via the perforin/granzyme or death receptor pathways^1,2^. NK cell target recognition is directed by integrating signals from a variety of NK inhibitory and activating receptors that control their response to a potential diseased cell^3–5^. Based on this unique immunobiology, NK cells are being developed as a cellular therapy for cancers and other diseases. NK cells express two T-box transcription factors (TF), T-bet and Eomes^6–8^. We recently used CRISPR/Cas9 loss-of-function approaches in human NK cells to demonstrate that sustained expression of Eomes and T-bet is required for human NK cell homeostasis, function, and identity^9^. However, the in vivo study of human NK cells lacking one or both of these T-box TF were limited to xenograft transfers using immunodeficient mice, which precludes an integrated study of the transferred NK cells in a species-matched environment.

In mouse NK cells, T-bet and Eomes have been shown to be critical for their development^10–12^. When Eomes expression is abrogated specifically in the hematopoietic compartment using Vav-Cre, NK cell numbers are reduced, and in global T-bet-deficient mice, NK cell frequency is significantly decreased. Since these previous models used hematopoietic and constitutive knockout approaches that led to compromised NK cell development, they do not support genetic loss-of-function studies of mature NK cells^7,10,11,13^. Hence, the requirement of these T-box TFs for mature NK cell homeostasis and function after development remains unclear. To address this gap in the field, we generated a tamoxifen-inducible *Ncr1*-iCreER^T2^ mouse that allows the study of conditional and inducible gene deletion in a fully developed, intact NK cell and ILC1 (NKp46^+^) compartment^14^. Our previous study was restricted to *Ncr1*-iCreER^T2^ x *Eomes*^f/f^ mice (Ncr1-Eomes^Δ/Δ^) and showed that sustained Eomes expression is important for NK cell function and homeostasis beyond promoting NK cell development. These findings caution against using broad T-box TF loss-of-function mouse models which have impaired NK development, non-NK/ILC effects, and/or ex vivo or adoptive transfer models, and highlight the importance of using conditional, inducible models to study the role of T-box TFs in mature NK cells^7,12,14,15^.

Studies of T-bet and Eomes have shown overlapping targets at the genomic level due to their highly homologous T-box DNA binding domain, but they have also been demonstrated to have unique functions^6,10,15–17^. In NK cell development, Eomes appears to be key for early NK maturation, whereas T-bet has been implicated as most important for terminal NK cell maturation^7,13^. Furthermore, T-bet is also involved in controlling NK cell egress from the bone marrow (BM)^18,19^. Here, we generate *Ncr1*-iCreER^T2^ x *Rosa26-*YFP^LSL^ x *Tbx21*^fl/fl^ (Ncr1-Tbx21^Δ/Δ^) mice to study inducible T-bet deletion in an intact NK compartment with fully developed and mature NK cells and other group 1 ILCs, which failed to develop in previous global and constitutive T-bet deletion mouse models^7,10,11^. Furthermore, we directly compare Ncr1-Tbx21^Δ/Δ^ and Ncr1-Eomes^Δ/Δ^ mice to discover the differential requirement for T-bet, Eomes, NK cells, and ILC1s for protection against murine cytomegalovirus (MCMV) infection.

Here, we use these genetic models to investigate the role of T-bet in the homeostasis and function of fully developed NK cells and group 1 ILCs using this Ncr1-conditional, inducible T-bet loss-of-function model. We find that loss of T-bet results in the tissue-specific loss of the most mature CD27^-^CD11b^+^ NK cells with sparing of the lymph nodes and bone marrow. Combined loss of both T-bet and Eomes results in a rapid, profound loss of all NK cells. When challenged with MCMV, we unexpectedly find that T-bet and ILC1s are dispensable for host protection, while Eomes and NK cells are required. These results highlight the fundamental role of T-box transcription factors in group 1 ILC homeostasis and function, and the importance of evaluating the effects of immune cell types using highly precise, temporally controllable genetic models of loss of function.

## Results

### Induced T-bet deletion results in alteration of NK cell compartment maturation stage distribution

**Fig. 1:**
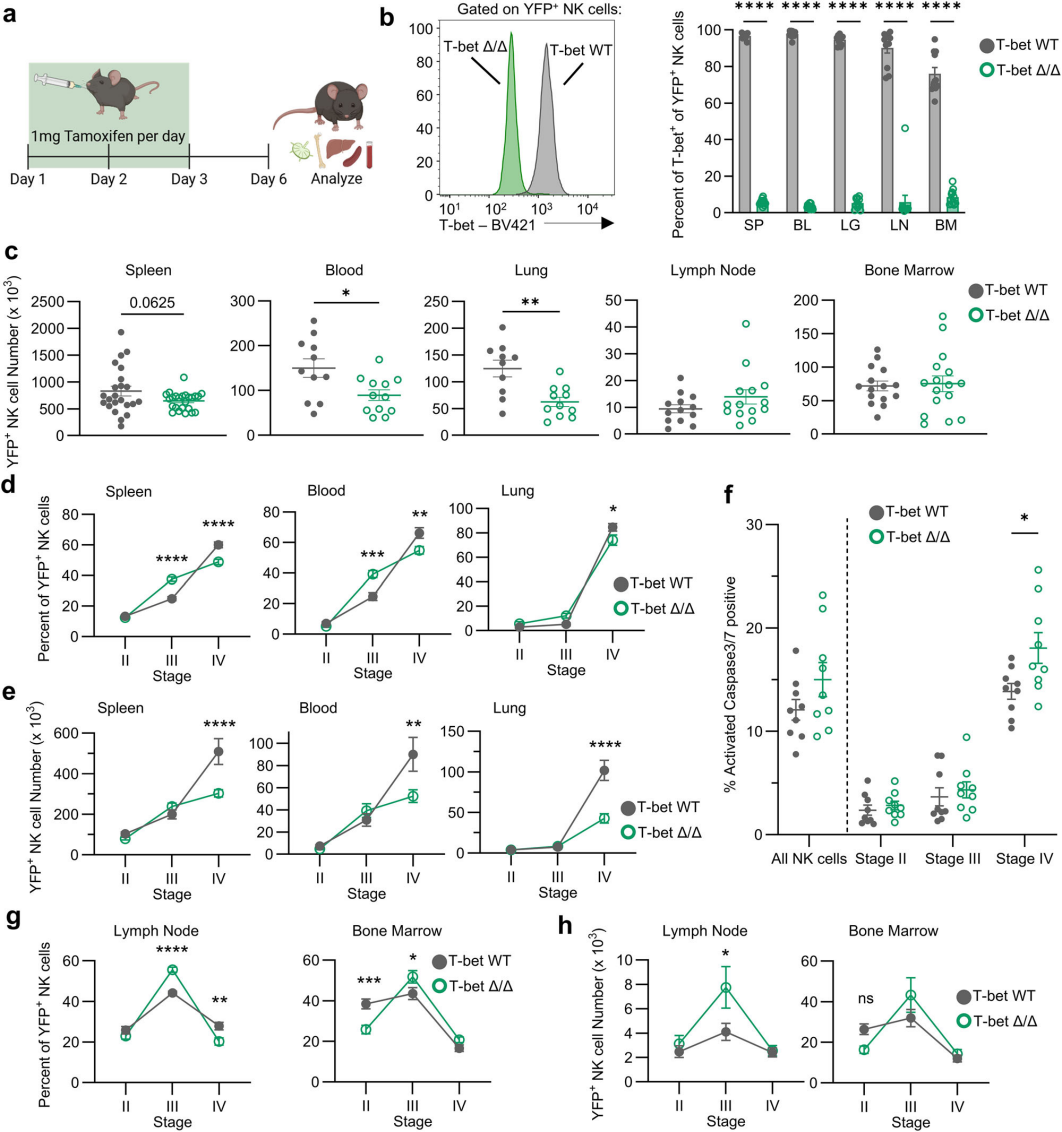
Induced T-bet deletion results in alteration of NK cell maturation. **a** *Ncr1*-iCreER^T2^ x *Rosa26-*YFP^LSL^ x *Tbx21*^fl/fl^ (T-bet Δ/Δ) mice and Ncr1-iCreER^T2^ x Rosa26-YFP^LSL^ x Tbx21^WT/WT^ (T-bet WT) mice were treated with 1 mg tamoxifen by oral gavage for 3 days (Day 1-3), then on Day 6, mice were analyzed. YFP^+^ percentage of NK cells (CD3^-^NK1.1^+^NKp46^+^) in indicated organs: spleen (SP), blood (BL – per mL), lung (LG), lymph nodes (LN – inguinal LNs), bone marrow (BM – per femur). Created in BioRender. Fehniger, T. (2026) https://biorender.com/phgwwq4. **b** Representative histogram of T-bet expression, left. T-bet^+^ percentage within YFP^+^ NK cells, right (*n* = 10-12 per group). **c** YFP^+^ NK cell numbers in the 5 different tissues indicated (*n* = 10-24 per group). **d, e** Summary data of percentage (*n* = 10-24 per group) and absolute numbers of YFP^+^ NK cells (*n* = 10-24 per group) within each indicated maturation stage. **f** assessment of activated Caspase 3/7 in YFP^+^ splenic NK cells (*n* = 9 per group). **g, h** Percentage (*n* = 13-16 per group) and absolute numbers (*n* = 13-16 per group) of YFP^+^ NK cells within each indicated maturation stage in the lymph node and bone marrow. Summary data are shown as mean ± SEM; Statistical significance was determined by multiple unpaired t-tests with Holm-Šídák method in **b** for adjusted p-values, two-sided unpaired t-tests in **c**, and by 2-way ANOVA with Šídák’s multiple comparisons test in (**d–h**) for adjusted p-values. 3-7 independent experiments. * = *p* < 0.05, ** = *p* < 0.01, *** = *p* < 0.001, **** = *p* < 0.0001. Exact p-values are provided in the Source Data file.

To assess the requirement for ongoing T-bet expression in a fully-developed and matured NK cell compartment, tamoxifen-inducible, NKp46^+^ cell specific, T-bet knock out mice (iCreER^T2^ x *Rosa26-*YFP^LSL^ x *Tbx21*^fl/fl^; Ncr1-Tbx21^Δ/Δ^) were generated (Supplementary Fig. 1a)^14,20^. To induce Cre-mediated recombination, these mice were treated with tamoxifen for 3 days by oral gavage (Day 1-3), and on Day 6 NK cell numbers and maturation were assessed (Fig. 1a). The inducible *Cre* activity was efficient with ~90% of NK1.1^+^NKp46^+^ cells expressing YFP, and T-bet protein expression was eliminated within these YFP^+^ NK cells consistently across various tissues (Fig. 1a, b, Supplementary Fig. 1b, c). The expression of Ly49 receptors, important for NK cell education and target cell recognition, was intact in Ncr1-Tbx21^Δ/Δ^ NK cells (Supplementary Fig. 1d). In the peripheral blood and lung, total NK cell numbers were significantly decreased. In contrast, in the spleen, lymph nodes (LN), and BM, NK cell numbers were similar within control and Ncr1-Tbx21^Δ/Δ^ mice (Fig. 1c).

We next assessed the impact of T-bet deficiency on NK cell maturation stages. After murine NK cells differentiate from an NK cell precursor, they undergo sequential maturation stages^21^. Stage I NK cells (CD27^-^CD11b^-^) are the most immature subset that are very low in frequency (Supplementary Fig. 1c). Stage II NK cells (CD27^+^CD11b^-^) are highly proliferative but have reduced effector functions compared to the more mature Stage III (CD27^+^CD11b^+^) and Stage IV (CD27^-^CD11b^+^) NK cells^21^. In Ncr1-Tbx21^Δ/Δ^ mice, Stage IV NK cells were selectively ablated in the spleen, blood, and lung while Stage II and Stage III NK cell numbers were unaffected on Day 6 (Fig. 1d, e). The loss of stage IV NK cells was also observed on Day 4, although reduced in magnitude compared to Day 6 (Supplementary Fig. 2b–d). Since the loss of stage IV NK cells was rapidly evident after tamoxifen-induced Cre activity, their loss seems unlikely to be due to a block in maturation but instead due to compromised survival. Indeed, assessment of activated Caspase 3/7 of Ncr1-Tbx21^Δ/Δ^ NK cells revealed evidence of increased apoptosis selectively within Stage IV (Fig. 1f). We considered evaluating the ability of stage-sorted NK cells for an impact of T-bet loss on maturation, but technical limitations of culturing murine NK cells and the lack of in vitro differentiation from Stage II/III to IV precluded these experiments (Supplementary Fig. 3). Interestingly, in LN and BM where the majority of NK cells are immature subsets, there was an increase in Stage III NK cells, while Stage IV NK cell numbers remain intact (Fig. 1g, h). Thus, induced T-bet deficiency in mature NK cells led to the loss of Stage IV NK cells by apoptosis in some tissues (spleen, blood, lung), whereas these cells in other tissues (LN, BM) remained resistant to T-bet deficiency.

### Cytotoxicity, but not IFN-γ production, is impaired following induced T-bet deletion

**Fig. 2:**
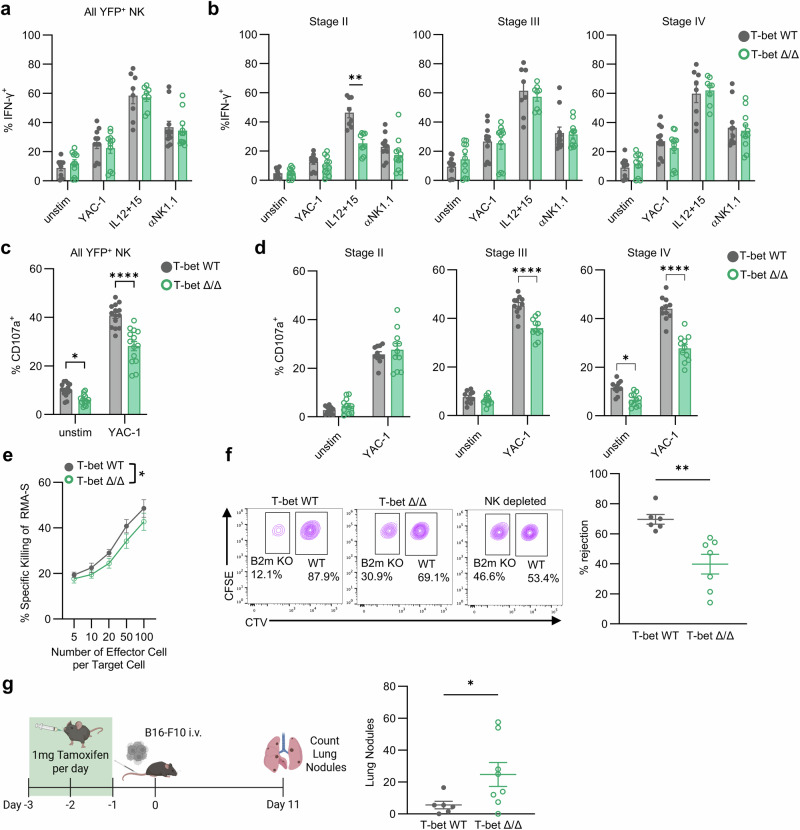
IFN-γ response is preserved but cytotoxicity is impaired in Ncr1-T-bet^Δ/Δ^ NK cells. **a–e***Ncr1*-iCreER^T2^ x *Rosa26-*YFP^LSL^ x *Tbx21*^fl/fl^ (T-bet Δ/Δ) mice were treated with 1 mg tamoxifen by oral gavage for 3 days (Day 1-3), then on Day 6, splenocytes were harvested for ex vivo functional and specific killing assessment. IFN-γ production by a, all YFP^+^ NK cells (CD3^-^NK1.1^+^NKp46^+^) (*n* = 8-11 per group) and b, YFP^+^ NK cells within indicated maturation stage in response to YAC-1, IL-12 + 15 and plate bound anti-NK1.1 stimulation (*n* = 8-11 per group). CD107a^+^ percentage within c, YFP^+^ NK cells (*n* = 14 per group) and d, YFP^+^ NK cells within the indicated maturation stage in response to YAC-1 stimulation (*n* = 14 per group). e, Specific killing of RMA-S by splenocytes (*n* = 10 per group). **f** 1:1 mixture of CFSE-labeled and cell trace violet-labeled splenocytes, B2m knock-out (KO) (CTVlo) and B2m wild-type (CTVhi), were injected into *Ncr1*-iCreER^T2^ x *Rosa*26-YFP^LSL^ x *Tbx21*^WT/WT^ (T-bet WT) or T-bet Δ/Δ mice on Day 4 of the tamoxifen regimen. Left, representative flow plots showing selective rejection of B2m KO splenocytes, and right, summary data of percent B2m KO rejection (*n* = 6–7 per group). **g** B16-F10 lung nodules 11 days after tumor cell inoculation (*n* = 6–8 per group). Created in BioRender. Fehniger, T. (2026) https://BioRender.com/e5csl1e. Summary data are shown as mean ± SEM; statistical significance was determined by 2-way ANOVA with Šídák’s multiple comparisons test in (**a**–**e**) for adjusted p values and determined by unpaired two-sided t-test with Welch’s correction in (**f**–**g**). 2–4 independent experiments. * = *p *< 0.05, ** = *p* < 0.01, *** = *p* < 0001, **** = *p *< 0.0001. Exact p-values are provided in the Source Data file.

Using the Ncr1-Tbx21^Δ/Δ^ model, we next determined whether T-bet is required for IFN-γ production by NK cells in response to target cells, cytokines, and activating receptor (NK1.1) engagement ex vivo (Fig. 2a, b, Supplementary Fig. 1g). As a bulk population, Ncr1-Tbx21^Δ/Δ^ NK cells produced similar IFN-γ as control NK cells (Fig. 2a), whether gating on NK1.1^+^NKp46^+^ or NK1.1^+^NKp46^+^CD49b^+^CD49a^-^ (Supplementary Fig. 4). Similarly, Ncr1-Tbx21^Δ/Δ^ NK cells had similar level of STAT4 phosphorylation in response to IL-12 stimulation (Supplementary Fig. 1e, f). However, when assessing individual maturation stages, Ncr1-Tbx21^Δ/Δ^ Stage II NK cells had significantly decreased IFN-γ specifically in response specifically to cytokines (IL-12 + IL-15 or IL-12 + IL-15 + IL-18) and comparable IFN-γ when triggered by tumor targets or NK1.1 ligation compared to control NK cells. In contrast, Stage III and IV NK cells had intact IFN-γ production (Fig. 2b, Supplementary Fig. 1h). These findings contrast previous studies in global T-bet deficient mice where all stages of NK cells had IFN-γ production impairment^13^. Instead, our NKp46-specific inducible T-bet deletion model indicates that mature NK cells do not require sustained T-bet to produce IFN-γ in response to typical NK cell-activating stimuli, and only the immature Stage II NK cells require T-bet for IFN-γ response to cytokines.

We next found NK cell degranulation (surface CD107a expression) in response to YAC-1 lymphoma target cells to be significantly reduced in Ncr1-Tbx21^Δ/Δ^ NK cells, specifically in Stage III and IV NK cells (Fig. 2c, d**;** Supplementary Fig. 4c), with a modest but significant decreased killing of RMA-S lymphoma by them (Fig. 2e). We utilized an in vivo MHC class I low (B2m^-/-^) splenocyte elimination assay to determine the in vivo impact of these changes within Ncr1-Tbx21^Δ/Δ^ mice (Fig. 2f). While T-bet WT mice selectively rejected B2m^-/-^ splenocytes, Ncr1-Tbx21^Δ/Δ^ mice had decreased ability to reject MHC-I deficient splenocytes (Fig. 2f). The inducible Ncr1-Tbx21^Δ/Δ^ mice also demonstrated impaired control of B16-F10 lung metastases, compared to control mice (Fig. 2g). Thus, selective loss of Stage IV NK cells in the blood and lung, along with decreased degranulation and killing, were sufficient to compromise control of B16-F10 metastases.

### Differential requirement of T-bet for group I ILCs in the liver

**Fig. 3:**
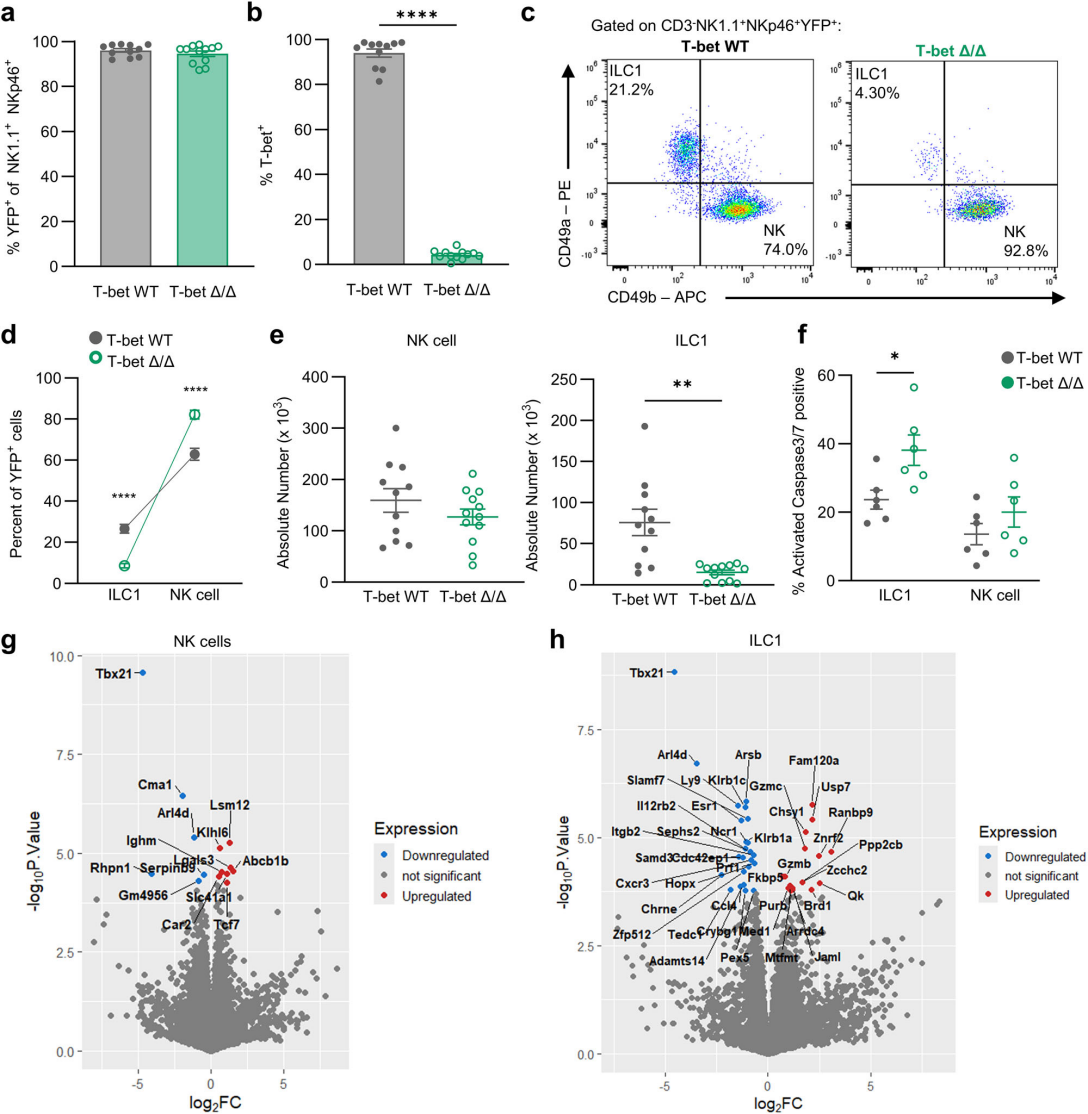
Liver ILC1s require sustained expression of T-bet to persist. *Ncr1*-iCreER^T2^ x *Rosa26-*YFP^LSL^ x *Tbx21*^fl/fl^ (T-bet Δ/Δ) and Ncr1-iCreER^T2^ x Rosa26-YFP^LSL^ x Tbx21^WT/WT^ (T-bet WT) mice were treated with 1 mg tamoxifen by oral gavage for 3 days (Day 1-3), then on Day 6, livers were harvested for analysis. **a** YFP^+^ percentage within all NK1.1^+^NKp46^+^ cells in the liver (*n* = 11-12 per group). **b** Percentage of T-bet^+^ cells within YFP^+^ NK1.1^+^NKp46^+^ cells in the liver (*n* = 11-12 per group). **c** Representative flow plots and **d**, summary percentages of YFP^+^ ILC1s (CD49a^+^CD49b^-^) and NK cells (CD49a^-^CD49b^+^) (*n* = 11-12 per group). **e** Absolute numbers of YFP^+^ NK cells and YFP^+^ ILC1s in the liver (*n* = 11-12 per group). **f** Apoptosis assessed by activated Caspase 3/7. 2-3 independent experiments (*n* = 6 per group). Summary data are shown as mean ± SEM; Statistical significance was determined by unpaired two-sided t-test with Welch’s correction in **a, b** and **e** and determined by 2-way ANOVA with Šídák’s multiple comparisons test in **c** and **f** for adjusted p values. * = *p* < 0.05, ** = *p* < 0.01, *** = *p* < 0.001, **** = *p* < 0.0001. Exact p-values are provided in the Source Data file. **g, h** T-bet Δ/Δ mice were treated with 1 mg tamoxifen by oral gavage for 2 days (Day 1-3), then on Day 3, liver g, NK cells and h, ILC1 from the same mice were flow sorted and used for RNA-sequencing. Genes with adjusted p-values ≤ 0.05 were colored to be upregulated (red) or downregulated (blue). *n* = 4 mice per group.

In addition to NK cells, ILC1s also express NKp46 and T-bet^1^. Previous studies with global T-bet knockout mice revealed that T-bet is required for the fetal development of ILC1 that become tissue-resident in the liver, as well as other NKp46^+^ ILC in the intestine^7,22^. However, whether a fully developed ILC1 compartment requires sustained T-bet expression for persistence is not known. To address this, we studied the NKp46^+^ T-bet^+^ ILC1 within Ncr1-Tbx21^Δ/Δ^ mice, where the Cre efficiency and T-bet ablation were highly efficient in NKp46^+^ cells in the liver, similar to other tissues (Fig. 3a, b). On Day 6 after tamoxifen-induced Cre recombination, we observed a loss of liver YFP+ stage IV NK cells, with no significant change in overall YFP^+^ NK cell numbers. In contrast, there was a marked reduction of ILC1 frequency and numbers in the liver (Fig. 3c–e, Supplementary Fig. 5a, b). This loss of ILC1 was already apparent earlier on Day 4 and persisted out to Day 22 after the tamoxifen (Supplementary Fig. 2e, Supplementary Fig. 7b, c). To investigate the mechanism of this loss, we performed activated Caspase 3/7 flow cytometry ex vivo on Day 4 after tamoxifen and observed significantly increased apoptosis of ILC1 (but not NK cells) in the liver (Fig. 3f). Similar to splenic NK cells, IFN-γ production in liver NK cells was mainly intact with the exception of Stage II NK cells that had significantly decreased IFN-γ in response to cytokines. In contrast, NK cell degranulation in response to YAC-1 lymphoma target cells was decreased, specifically in stage III and IV NK cells, similar to splenic NK cells (Supplementary Fig. 5c–f).

In terms of transcriptional remodeling from induced T-bet deficiency, bulk RNA-sequencing of liver NK cells in Ncr1-Tbx21^Δ/Δ^ mice revealed minimal transcriptional changes in NK cells at Day 3 of tamoxifen (Fig. 3g), consistent with an intact liver NK cell compartment. However, T-bet-deficient ILC1 exhibited significant gene expression changes, including genes encoding integrin and chemokine (*Cxcr3, Itgb2, Ccl4*), ILC activating receptors (*Ncr1, Klrb1c*), granzymes, as well as downregulation of the TF *Hopx* (Fig. 3g, h). Collectively, these data support that sustained T-bet expression is required for liver ILC1 (but not NK cell) homeostasis as Ncr1-Tbx21^Δ/Δ^ mice have a profound loss of ILC1 shortly after Cre induction, while liver NK cells remain intact at this time point. Further, the mature ILC1 transcriptional program is highly regulated by ongoing T-bet expression.

### Small intestine lamina propria NKp46^+^ ILC3s require sustained T-bet expression to persist

**Fig. 4:**
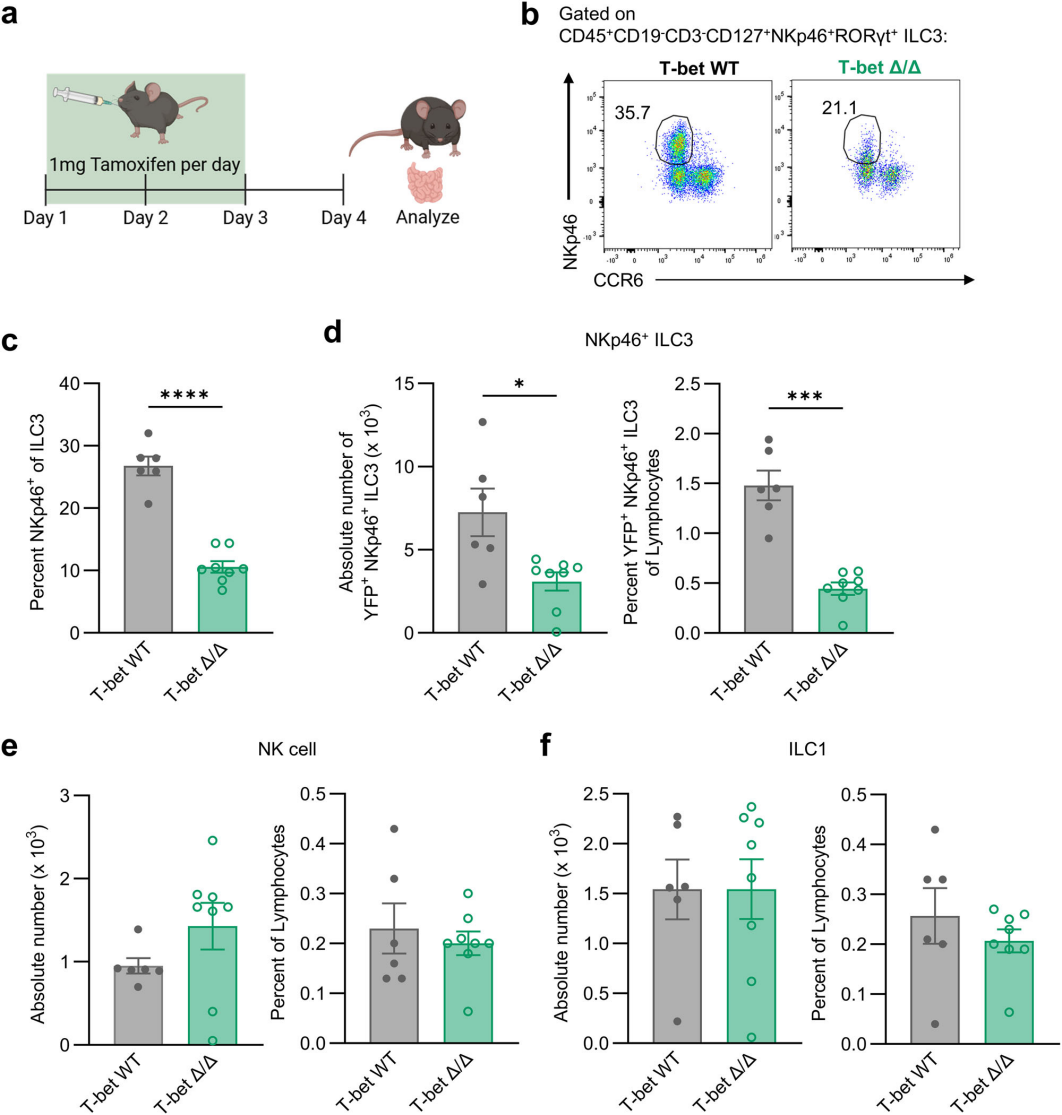
Small intestine lamina propria ILC3s, but not NK cells and ILC1s, require sustained expression of T-bet to persist. **a***Ncr1*-iCreER^T2^ x *Rosa26-*YFP^LSL^ x *Tbx21*^fl/fl^ (T-bet Δ/Δ) and Ncr1-iCreER^T2^ x Rosa26-YFP^LSL^ x Tbx21^WT/WT^ (T-bet WT) mice were treated with 1 mg tamoxifen by oral gavage for 3 days (Day 1-3), then on Day 6, mice were harvested for analysis. Created in BioRender. Fehniger, T. (2026) https://BioRender.com/87xmq1a. **b** Representative flow plot of NKp46^+^ ILC3s in the lamina propria of T-bet WT and T-bet Δ/Δ mice. **c** Summary data of YFP^+^ NKp46^+^ ILC3 percentage within all ILC3 (*n* = 6-8 per group). **d**–**f** Percentage of lymphocyte and absolute numbers of d, YFP^+^ NKp46^+^ ILC3 (*n* = 6-8 per group), e, YFP^+^ NK cells (*n* = 6-8 per group), and f, YFP^+^ ILC1 (*n* = 6-8 per group) in the small intestine lamina propria. 3 independent experiments. Summary data are shown as mean ± SEM; Statistical significance was determined by unpaired two-tailed t-tests with Welch’s correction in (**c**–**f**). * = *p* < 0.05, ** = *p* < 0.01, *** = *p* < 0.001, **** = *p* < 0.0001. Exact p-values are provided in the Source Data file.

NKp46^+^ ILC are also present in the small intestine lamina propria (SI LP), where in addition to ILC1 and NK cells, a subset of SI LP ILC3 express both NKp46 and T-bet (Supplementary Fig. 6a). We confirmed that induced T-bet deletion is highly efficient in NKp46^+^ cells in the SI LP, similar to other tissues (Supplementary Fig. 6b). Acute loss of T-bet expression over 6 days reduced numbers of NKp46^+^ ILC3 (but not NKp46^-^CCR6^+^ ILC3 and ILC2) in the SI LP (Fig. 4a–d, Supplementary Fig. 6c, d). Both SI LP NK cell and ILC1 numbers were not significantly altered despite confirmed T-bet deletion (Fig. 4e, f). However, persistent loss of T-bet over 22 days also led to a reduction of SI LP ILC1 (Supplementary Fig. 7g). The resistance of T-bet^+^ SI LP ILC1 to acute T-bet deletion is intriguing and contrasts with the rapid elimination of liver ILC1. Collectively, these data show that T-bet^+^ ILC3 in the SI LP requires ongoing T-bet for their homeostasis, and that ILC1 has a tissue-specific dependency for sustained T-bet expression.

### Sustained T-bet and Eomes are required for NK cell homeostasis in peripheral tissues

**Fig. 5:**
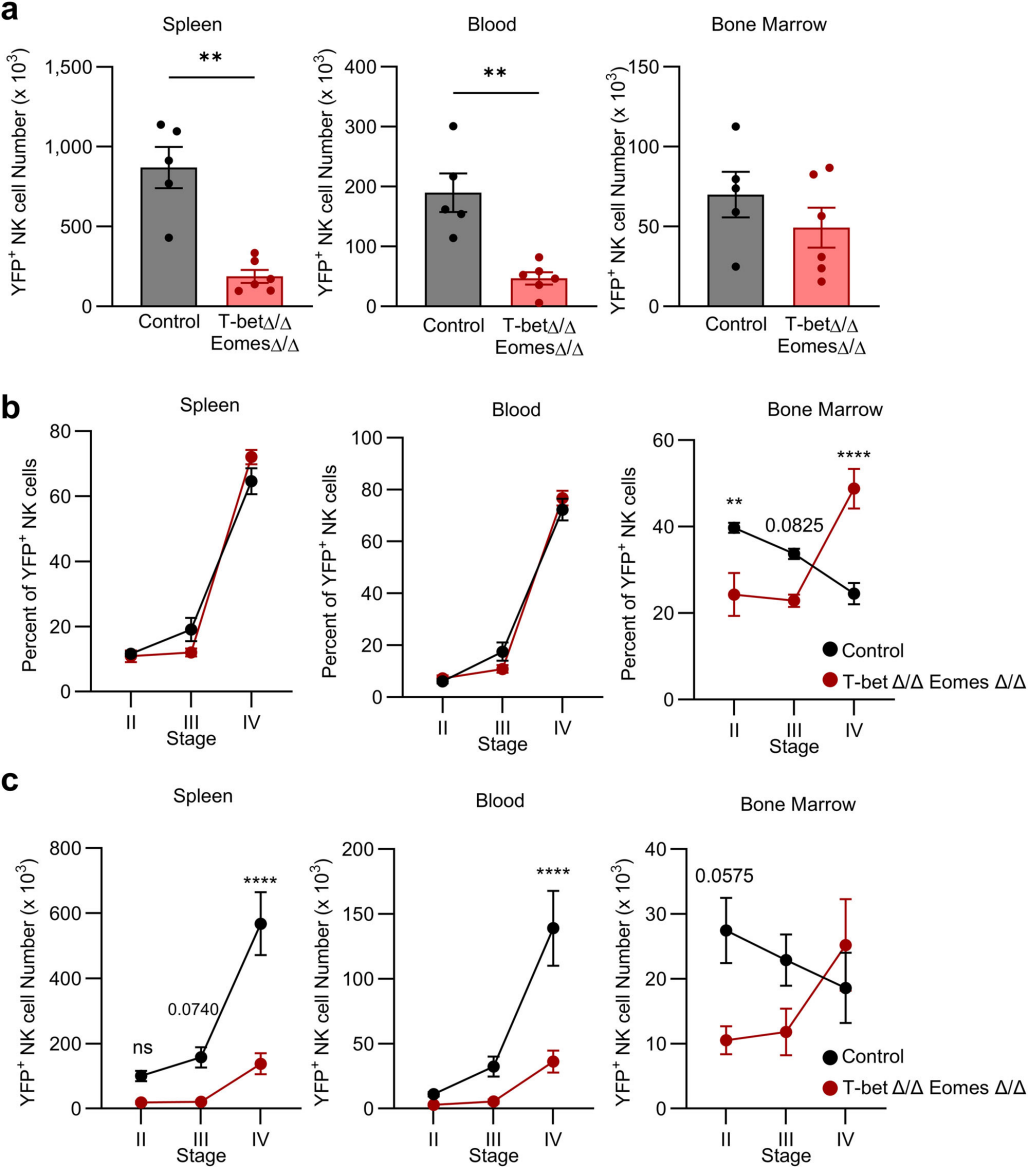
Inducible deletion of both T-bet and Eomes led to loss of NK cells in the spleen and blood but does not affect Stage IV NK cells in the bone marrow. *Ncr1*-iCreER^T2^ x *Rosa26-*YFP^LSL^ x *Tbx21*^fl/fl^ x *Eomes*^Δ/Δ^ (T-betΔ/Δ EomesΔ/Δ) or *Ncr1*-iCreER^T2^ x *Rosa26*-YFP^LSL^ (control) mice were treated with 1 mg per day tamoxifen by oral gavage for 3 days (Day 1-3), then on day 3-4 mice were analyzed. **a** Summary data of all YFP^+^ NK cells (CD3^-^NK1.1^+^NKp46^+^) numbers in the spleen, blood, and bone marrow (BM) (*n* = 5-6 per group). Summary data of **b** percentage (*n* = 8-9 per group) and **c** absolute numbers of YFP^+^ NK cells within each indicated maturation stage on Day 3-4 (*n* = 8-9 per group). 3 independent experiments. Statistical significance was determined by an unpaired two-tailed t-test with Welch’s correction in **a** and a 2-way ANOVA with Šidák’s multiple-comparisons test in (**b, c**) for adjusted p-values.* = *p* < 0.05, ** = *p* < 0.01, *** = *p* < 0.001, **** = *p* < 0.0001. Exact p-values are provided in the Source Data file.

Previous studies in human NK cells and murine models have suggested that T-bet and Eomes have overlapping functions^7,9^. To address the potential redundancy of T-bet and Eomes, we generated Ncr1-specific, inducible knockout mice that lack both T-box TFs (Ncr1-Tbx21^Δ/Δ^Eomes^Δ/Δ^). After tamoxifen-induced deletion of T-bet and Eomes, there was a marked loss of NK cells that was observed as early as Day 3-4 in the spleen and blood (Fig. 5a). All NK cells regardless of maturation stage were affected by the loss of T-bet and Eomes, but the greatest magnitude of effect was in Stage IV NK cells. Surprisingly, total NK cell numbers were unaltered in the BM of Ncr1-Tbx21^Δ/Δ^Eomes^Δ/Δ^ mice, even up to Day 9 after tamoxifen (Fig. 5a, Supplementary Fig. 8). In the BM, Stage IV NK cell numbers were not significantly different in Ncr1-Tbx21^Δ/Δ^Eomes^Δ/Δ^ mice, while Stage II and Stage III NK cell frequency and numbers were decreased (Fig. 5b, c). These data suggest that loss of T-box TFs profoundly impacts most NK cells, with the exception of mature NK cells residing in the BM.

### Individual contribution of T-bet and Eomes within group 1 ILC during MCMV infection

**Fig. 6:**
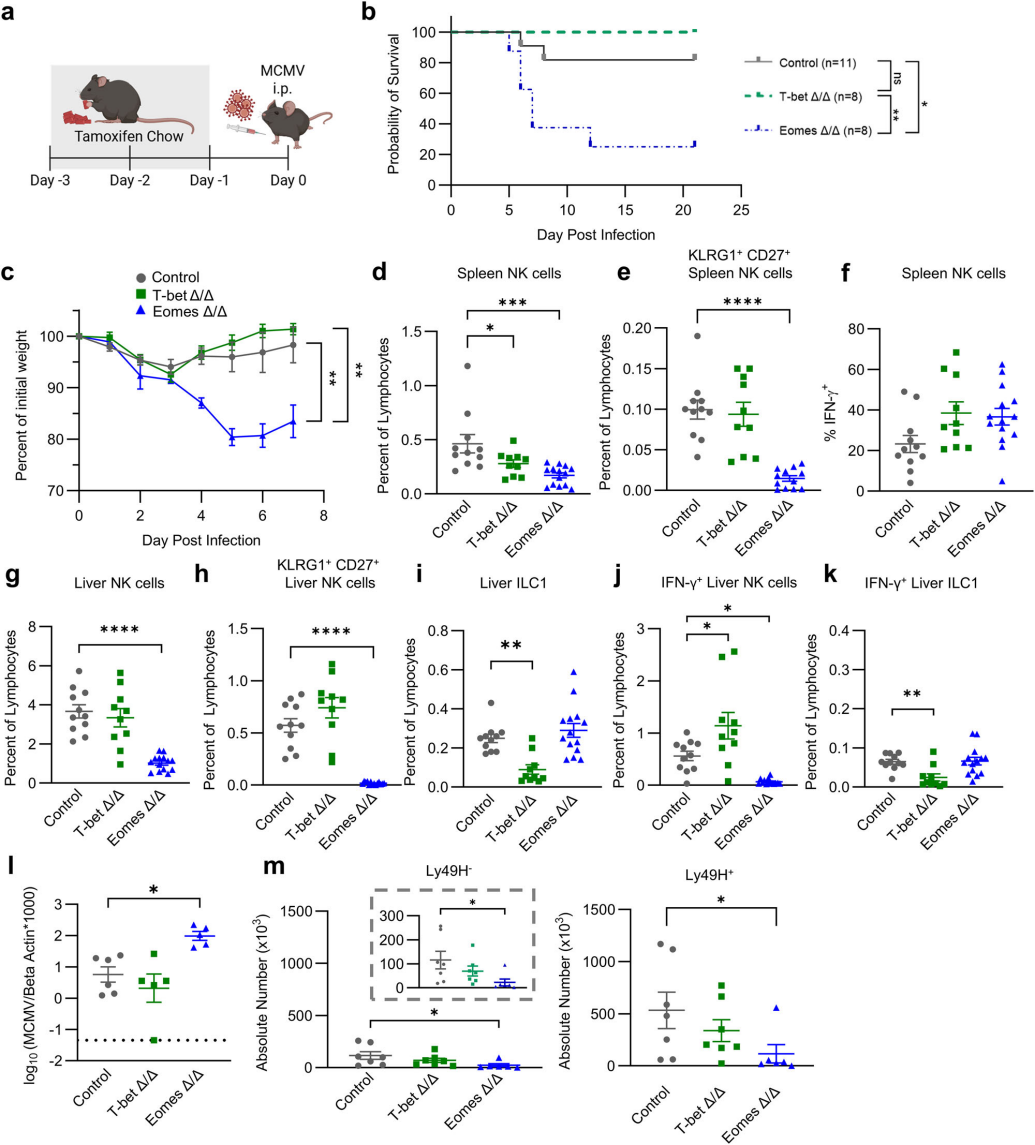
T-bet is dispensable while Eomes is required for protective group 1 ILC mediated murine cytomegalovirus (MCMV) response. **a** Schema. *Ncr1*-iCreER^T2^ x *Rosa26-*YFP^LSL^ x *Tbx21*^fl/fl^ (T-bet Δ/Δ), *Ncr1*-iCreER^T2^ x *Rosa26-*YFP^LSL^ x *Eomes*^fl/fl^ (Eomes Δ/Δ), and N*cr1*-iCreER^T2^ x *Rosa26*-YFP^LSL^ (control) mice were fed tamoxifen chow for 3 days. The day after tamoxifen chow was removed, the mice were infected with **b**, 2 ×105 pfu or (c-m) 5 ×104 pfu MCMV. Schema created in BioRender. Fehniger, T. (2026) https://BioRender.com/x3jcenb. **b** Kaplan-Meier survival curve (*n* = 8–11 per group, female mice only). **c** Summary weigh loss data (*n* = 4–6 per group). Summary data of **d**, YFP^+^ NK cell (CD3^-^NK1.1^+^NKp46^+^) percentage within splenic lymphocytes (*n* = 10–14 per group), **e** KLRG1^+^CD27^+^ YFP^+^ NK cells percentage within splenic lymphocytes (*n* = 10–12 per group), and **f** percentage of IFN-γ^+^ splenic YFP^+^ NK cells (*n* = 10–14 per group) at 1.5 days post MCMV infection. **g**–**i** percent of g, YFP^+^ NK cell (*n* = 10-14 per group), h, KLRG1^+^CD27^+^ YFP^+^ NK cell (*n* = 10–14 per group), and i, ILC1 within lymphocytes in the liver (*n* = 10–14 per group). **j**, **k** percent of IFN-γ^+^ NK cell (*n* = 10-14 per group) and ILC1 (*n* = 10-14 per group) within lymphocytes in the liver. l, MCMV titer measured as IE1 copy number per Beta actin *1000 in the spleen on 4 days post infection (*n* = 5–6 per group). **m** Absolute numbers of Ly49H^-^ and Ly49H^+^ NK cells in the spleen 7 days post infection (*n* = 6–7 per group). Statistical significance was determined by Mantel-Cox test in **b**, Mixed-effect analysis with Šidák’s multiple comparison test in c for adjusted p-values, and one-way ANOVA with Dunnett’s multiple-comparisons test in d-l and Kruskal-Wallis test with Dunn’s multiple comparisons in m for adjusted p-values. 2–4 independent experiments. * = *p* < 0.05, ** = *p* < 0.01, *** *p* = < 0.01, **** = *p* < 0.0001. Exact p-values are provided in the Source Data file.

A previous study has indicated that both T-bet and Eomes are important for the expansion of NK cells during MCMV infection using an adoptive transfer model^15^. Here, we treated Ncr1-Tbx21^Δ/Δ^ or Ncr1-Eomes^Δ/Δ^ mice with tamoxifen chow for 3 days and then infected them with a sublethal dose of MCMV on day 4. When infected with 2×10^5^ pfu MCMV (LD_20_), there was an approximately 75% mortality in Ncr1-Eomes^Δ/Δ^ mice, contrasting to 0% mortality in the Ncr1-Tbx21^Δ/Δ^ (Fig. 6a, b). To elucidate the mechanism of this loss of protection by Ncr1-Eomes^Δ/Δ^ and intact host defense within Ncr1-Tbx21^Δ/Δ^ mice, we infected mice with a lower (sub-lethal) dose of 5 x 10^4^ pfu MCMV. At this lower dose, all mice lost weight during the course of infection as a measure of MCMV mediated morbidity, but Ncr1-Eomes^Δ/Δ^ mice had significantly more weight loss, also supporting that induced lack of Eomes in NK cells results in a compromised response against MCMV (Fig. 6c). Assessment of the spleen at 1.5 days post infection show that NK cell frequency was decreased in Ncr1-Tbx21^Δ/Δ^ and Ncr1-Eomes^Δ/Δ^ mice compared to control (Fig. 6d). Notably, NK cells that express both KLRG1 and CD27, both of which are induced during MCMV infection, are markedly and selectively reduced in Ncr1-Eomes^Δ/Δ^ (Fig. 6e). Moreover, despite this alteration in NK cell numbers and activation in Ncr1-Eomes^Δ/Δ^ mice, IFN-γ production, critical for early host response to MCMV response, by the few remaining splenic YFP^+^ NK cells was intact (Fig. 6f)^23^.

We next assessed the group 1 ILC compartment in the liver at 1.5 days post-infection, where MCMV infection occurs. NK cell frequency was significantly decreased in Ncr1-Eomes^Δ/Δ^ mice with near complete loss of the activated KLRG1^+^CD27^+^ NK population (Fig. 6g, h). However, in contrast to the spleen, Ncr1-Tbx21^Δ/Δ^ mice did not have reduced NK cell frequency in the liver, but exhibited a significantly reduced ILC1 frequency (Fig. 6g–i). The small number of residual Ncr1-Tbx21^Δ/Δ^ ILC1 were able to produce IFN-γ similar to ILC1s from control mice; NK cells from Ncr1-Tbx21^Δ/Δ^ mice had increased IFN-γ production, while the few residual Ncr1-Eomes^Δ/Δ^ NK cells produced significantly less IFN-γ compared to control mice (Supplementary Fig. 9a). Collectively, the total frequency of IFN-γ producing NK cells, but not ILC1, were severely decreased in Ncr1-Eomes^Δ/Δ^ mice compared to control mice (Fig. 6j, k). This is consistent with significantly higher viral burden in the spleen that persisted at 4 days post infection in the Ncr1-Eomes^Δ/Δ^ mice compared to Ncr1-Tbx21^Δ/Δ^ and control mice (Fig. 6l, Supplementary Fig. 9b, c).

We also investigated Ncr1-Tbx21^Δ/Δ^ and Ncr1-Eomes^Δ/Δ^ NK cells 7 days post MCMV infection, focusing our assessment on the activated MCMV-specific Ly49H^+^ NK cell compartment, that recognizes m157 on MCMV-infected cells. NK cells that had T-bet or Eomes deleted had similar Ly49H expression (Supplementary Fig. 9d). The absolute number of Ly49H^-^ and Ly49H^+^ splenic NK cells on day 7 post infection were significantly decreased in Ncr1-Eomes^Δ/Δ^, but not Ncr1-Tbx21^Δ/Δ^ mice (Fig. 6m). This loss of NK cells within Ncr1-Eomes^Δ/Δ^ mice was likely explained by increased apoptosis (Fig. 6e, Supplementary Fig. 9e). At this time point, Ly49H^+^ cells were primarily stage IV, and we observed a significant decrease in their numbers within both Ncr1-Tbx21^Δ/Δ^ and Ncr1-Eomes^Δ/Δ^ mice (Supplementary Fig. 9f). Thus, inducible Ncr1-Tbx21^Δ/Δ^ and Ncr1-Eomes^Δ/Δ^ models revealed that T-bet within the NKp46^+^ compartment is not required for MCMV protection. These models also reveal that within the Ncr1-Tbx21^Δ/Δ^ mice ILC1 frequency were markedly reduced, yet they had normal host protection against MCMV. In contrast, Ncr1-Eomes^Δ/Δ^ mice had a marked defect in only their NK cell compartment, with intact liver ILC1 populations, yet were unable to produce an effective, protective MCMV response, implicating NK cells as the critical group 1 ILC protecting the host in early stages of infection.

## Discussion

Here we developed an inducible, conditional Ncr1-Tbx21^Δ/Δ^ model to study the requirements for ongoing T-bet expression within NKp46^+^ ILCs and its impact on homeostasis and immunobiology. We demonstrated that persistent T-bet expression is required to sustain 1) the mature NK cell compartment in the spleen and peripheral tissues, 2) ILC1 in the liver, and 3) NKp46^+^ ILC3 in the small intestine lamina propria. Moreover, T-bet was also required for normal NK cell killing of MHC-I-deficient targets and lung metastases. Surprisingly, T-bet was largely dispensable for IFN-γ production by mature NK cells after in vitro stimulation, and Ncr1-Tbx21^Δ/Δ^ mice had an intact NK cell response to MCMV infection in vivo. By contrasting Ncr1-Tbx21^Δ/Δ^ mice to Ncr1-Eomes^Δ/Δ^ mice during MCMV infection, we identified a critical role for Eomes and NK cells in host defense against MCMV, while T-bet and ILC1 were not required. Additionally, induced T-bet and Eomes genetic deletion after normal group 1 ILC development using the Ncr1-CreER^T2^ model led to a rapid and profound loss of NK cells, indicating a non-redundant role for T-box TFs to maintain these ILC populations.

Determining the requirement for T-box TFs in mature ILCs was challenging in previous global and constitutive genetic deletion mouse models, since NK cell and ILC1 populations were absent due to lack of development^7,11–13^. Previous studies using global T-bet knock-out models, where the effect of T-bet deficiency in other (non-NKp46^+^) cells was a confounding factor, reported reduced NK1.1^+^ cell numbers in the spleen, liver, and blood^7,11,13^. Here, when T-bet is deleted in mature NK cells, only Stage IV (CD27^-^CD11b^+^) NK cells in the spleen and blood were compromised. Our data suggests that this is due to increased apoptosis; however, differentiation may also play a role. Further, in the liver there was a marked loss of ILC1s in the Ncr1-Tbx21^Δ/Δ^ mice while only Stage IV NK cells were decreased. Another prior global T-bet knockout study concluded that NK cells have a trafficking defect^18^. Here, we show that mature NK cell survival in the blood requires NK cell-intrinsic T-bet, which may have contributed to reduced NK cells in the blood observed in the global T-bet knockout. These examples highlight the importance of employing inducible, conditional Cre models to fully understand the impact of developmentally required TF on immune cell homeostasis and function.

Examining mature NK cells for the first time following induced combined T-box TFs genetic loss, our findings provide the insight that NK cells absolutely require ongoing T-box TF expression to persist in the spleen and periphery after development. Gordon et. al. constitutively deleted T-bet and Eomes and observed that NK cells were lost both in the spleen and BM^7^. In contrast, our inducible T-bet and Eomes-deletion model revealed that these T-box TFs were required for spleen and peripheral NK cell homeostasis, but surprisingly not for NK cells in the BM. We hypothesize that the BM microenvironment is unique, supporting NK cell homeostasis via alternative pathways, such as via accessory cell populations that provide cell-contact dependent and/or secreted factors, including IL-15 the key survival cytokine for NK cells^24–27^. When NK cells are not in an environment with high abundance of extrinsic survival signals, T-box TFs may take on a critical role to maintain NK cell identity and survival. Notably, T-box TF expression levels have previously been shown to be lower in NK cells in the BM, compared to those in the periphery^10^, which also suggests the potential of other TFs supporting BM NK cells^10^. Perhaps TFs that are already expressed in NK cell progenitors and continued to be expressed by the immature NK cells in the BM (e.g., Ets1, Tcf7, Nfil3), are sufficient to support BM NK cells even when T-bet and Eomes expression is completely removed^28–31^. Future studies contrasting the genetic deletion of these candidate TFs are required to fully understand the transcriptional regulation of developing BM localized group 1 ILCs. In addition, many lung NK cells are vascular-associated and mimic the phenotype of blood NK cells; in future studies, CD45 labeling can be used to better define the impact of induced T-bet loss on lung parenchymal NK cells.

In Zhang et al. the investigators evaluated the relative expression of T-bet and Eomes using global T-bet^-/-^ mice and constitutive Ncr1-specific Eomes deletion in a landmark study^13^. In WT mice, T-bet and Eomes demonstrated an inverse pattern of expression across maturation stages with Eomes enriched in immature NK cells and T-bet enriched in mature NK cells. Contrasting gene expression between NK cells that arose in global T-bet^-/-^ to WT mice, Zhang et al. also identified IL-12 signaling genes, including the IL-12 receptor, as requiring T-bet for expression. Indeed, NK cells from global T-bet^-/-^ mice had reduced pSTAT4 signaling in response to IL-12, and reduced IFN-γ production in response to IL-12 cytokine combinations in all stages of NK cell maturation, yielding the conclusion that T-bet was required for IL-12 responsiveness. In contrast, in our model where NK cells and ILC1s mature normally and then have induced, Ncr1-specific loss of T-bet, we observed few changes in NK cell gene expression when T-bet was lost, in marked contrast to ILC1’s which had extensive gene expression changes. Further, in NK cells, we observed no significant alterations in IL-12 signaling pathway genes by RNA sequencing and no significant alterations in pSTAT4 by an IL-12 stimulation assay. IFN-γ production was intact in most NK cells, with only cytokine-induced IFN-γ reduced in Stage II NK cells. Thus, our study reveals that T-bet is required for IL-12 responsiveness during development and is dispensable for most mature NK cell IL-12 responses.

T-bet and Eomes have been previously studied in the context of MCMV host response. In a study from Madera et al.^15^, divergent roles for T-bet in effector and MCMV-memory NK cells were reported using adoptive transfer of NK cells, with T-box TF expression controlled via a global Ubiquitin-ER^T2^ Cre. Using this approach, minimal homeostatic changes in induced T-bet-deficient NK cells were reported. In contrast, our genetic model that eliminates T-bet expression in NK cells “in situ” shows a clear defect in the number of Stage IV NK cells. Additionally, Madera et al. reported that T-bet is required for MCMV memory NK cells utilizing an experimental system of adoptive NK cell transfer 3 weeks prior to MCMV infection. In this setting, the transferred NK cells differentiate in vivo into Stage IV NK cells, becoming the vast majority of transferred NK cells^21^. Thus, the apparent requirement for T-bet in this MCMV-memory experiment may not be specific to memory cells, and instead reflects the selective impact on Stage IV NK cells. In addition, while Madera et. al. also reported an increase in Ly49H expression on T-bet-deficient NK cells after adoptive transfer, we observed no significant change in NK cells that lost T-bet “in situ”. The previous study’s adoptive transfer setting indicated a requirement for both T-bet and Eomes for the initial response to MCMV, while our study showed that induced T-bet loss did not dampen the initial response to MCMV. Instead, only induced Eomes loss resulted in marked susceptibility to MCMV infection. Together, these findings highlight the importance of considering the physiological context (e.g., adoptive transfer) and timing of T-box TF deletion to assess requirements for in vivo NK cell responses.

Weizman et al. reported that ILC1s are required during early MCMV infection for host protection^28^, using a global Zfp683 (Hobit) deficient mice to model ILC1 deficiency. MCMV was highly lethal to the global Zfp683^-/-^ mice, however, Hobit deficiency in other immune cell types may contribute to host defense against MCMV. For example, Hobit is also upregulated by T cells during infection and modulates T cell tissue residency, which may contribute to the anti-MCMV response^29^. In our study, conditional, inducible Ncr1-Tbx21^Δ/Δ^ mice rapidly developed a profound ILC1 deficiency, yet host protection from MCMV was intact. Survival and MCMV titers at day 4 post-infection were not different in Ncr1-Tbx21^Δ/Δ^ compared to control mice. This agrees with a study by Yomogida et al., using constitutive Ncr1-specific Hobit deletion mice that abrogated ILC1 development. No MCMV titer difference were observed compared to control mice at 7 days post MCMV infection (survival data was not reported in this study)^30^. Thus, our data indicate that despite ILC1’s rapidly producing IFN-γ during early MCMV infection (as demonstrated by Weizman et al.), they are not required for normal MCMV host defense in the conditions used in our experiments^28^. This likely reflects the relatively low frequency of ILC1 compared to NK cells, abundant NK cell-IFN-γ at 36 h post-infection, higher NK cell frequency at the sites of MCMV infection (spleen and liver), and robust proliferation over the course of the infection.

Other tissue-resident ILCs express NKp46 and T-bet, and analysis of Ncr1-Tbx21^Δ/Δ^ mice revealed that mature liver ILC1 and SI LP ILC3 depend on the persistent expression of T-bet for survival. This indicates that T-bet provides ongoing transcriptional regulation that was not compensated for by other ILC1- or ILC3-associated TFs such as Hobit and RORγt, respectively. Our observation that ILC1s in the SI LP, unlike those in the liver, were not rapidly affected by the loss of T-bet agrees with recent studies that show the tissue-specific differences between ILC subsets, and raises new avenues of investigation^30–32^. In Schroeder et al., the authors also describe the differential requirement of T-bet in the maintenance of NKp46^+^NK1.1^+^ ILC1 in colonic and SI LP vs. intraepithelial lymphocyte (IEL)^33^. Factors in the SI LP that allow ILC1s to persist without T-bet warrant further studies to reveal the dominant TF(s) and/or alternative signals that govern their survival.

As NK cell-based therapies emerge for cancer, autoimmunity, and infectious diseases, the need to understand NK cell immunobiology in various contexts beyond homeostasis is important^34–39^. In human NK cells, studies of Eomes and T-bet have been predominantly limited to peripheral blood NK cells. In agreement with our current mouse study, double knock-out of T-bet and Eomes was profoundly detrimental to human PB NK cell homeostasis and function^9^. However, it remains unclear what the relative expression of T-box transcription factors is within tissue resident^40,41^ and tumor resident^42–44^ human NK cell and ILC populations, and how they depend on the T-box transcriptome program for homeostasis. The tissue-specific phenotypes of mouse NK cells and ILCs reported here highlight this issue and the need for further investigation in the setting of human disease and NK cell-based immunotherapy.

## Methods

### Mice

*Ncr1*-iCreER^T2^ x *Rosa26-*YFP^LSL^ and *Ncr1*-iCreER^T2^ x *Rosa26-*YFP^LSL^ x *Eomes*^fl/fl^ (Ncr1-Eomes^Δ/Δ)^ mice were generated by our lab as reported previously^14^. Here we cross *Ncr1*-iCreER^T2^ x *Rosa26-*YFP^LSL^ to *Tbx21*^fl/fl^ mice (Ncr1-T-bet^Δ/Δ^) (RRID:IMSR_JAX:022741). For all experiments, *Ncr1*-iCreER^T2 KI/WT or KI/KI^ x *Rosa26*-YFP^LSL^ x *Tbx21*^*wt/wt*^ mice were used as controls unless otherwise indicated. Experimental and control animals were bred and maintained in the same specific pathogen-free facility with a standard 12 h light/12 h dark cycle at 30-70% relative humidity at ambient temperature at Washington University. Animals were euthanized by CO_2_ asphyxiation followed by cervical dislocation, following WashU IACUC guidelines.

Tamoxifen was administered in corn oil (Sigma, T5648) at 1 mg per day up to 3 days via oral gavage. For Fig. 6, tamoxifen chow (Envigo TD.130855) was used instead of oral gavage. Mice were bred and maintained in specific pathogen-free housing, and experiments were conducted in accordance with the guidelines of and with the approval of the Washington University Institutional Animal Care and Use Committee (20170129, 23-0200). Experiments were performed using mice between 7–20 weeks of age. Animals were age-matched within approximately 2 weeks for each experiment. Both sexes of mice were used in this study. Specific numbers, sex(es), and age range are indicated within the Source Data file for each figure. For mouse survival experiments, mice were euthanized following WashU IACUC guidelines if they lost more than 20% of their body weight, displayed paralysis, or were moribund.

### Cell lines

YAC-1 (CVCL_2244, ATCC TIB-160) and RMA-S (RRID:CVCL_2180, kind gift from Yokoyama lab) were maintained at 37 °C in 5% CO_2_ in RPMI-1640 plus 10% FBS and supplements; B16-F10 (RRID:CVCL_0159, ATCC CRL-6475) cells were maintained in DMEM plus 10% FBS and supplements following ATCC guidelines and kept in continuous culture for < 2 months.

### Organ isolation and processing

Mice were sacrificed by CO_2_ asphyxiation and organs harvested immediately. Cardiac puncture was performed to collect blood prior to removal of other organs when appropriate. Spleens and inguinal lymph nodes were isolated and crushed through a 70 µm filter to generate a single cell suspension. Lungs were first digested with collagenase (Sigma, C9407) and DNase I (Sigma, D4527) prior to further processing. BM was harvested by flushing one femur from each mouse with a 23-gauge needle. Whole livers were pulverized with glass douncers followed by percoll gradient to isolate lymphocytes. All tissues were ACK lysed to remove red blood cells. Single cell suspensions were plated for subsequent flow cytometry staining and functional assessments. Activated caspase 3/7 was determined using FLICA Caspase-3/7 Assay Kit (Immunochemistry Technologies 9125)

### Flow cytometry and cell sorting

Surface antibody staining was performed as previously described^14^. Intracellular staining was performed in permeabilization buffer following fixation/permeabilization with kits from BD Biosciences (for IFN-γ staining, BD 554722) or Invitrogen eBioscience Fix/Perm kit (Thermo Fisher, 00-5123-43, 00-5223-56). When using the eBioscience Fix/Perm kit which quenches YFP, after surface staining cells were briefly fixed in 1% PFA for 2 minutes at room temperature prior to fixation/permeabilization in order to preserve YFP when intracellular proteins were assessed. For T-box TF staining, cells were incubated in eBioscience 1X Fix/Perm overnight at 4 °C in the dark, then the next day proceeded to staining overnight in 1X Perm Buffer at 4 °C in the dark. Flow cytometry data were collected on Gallios, Attune Nxt, or Cytek Northern Light flow cytometers, and analyzed using FlowJo v10.8.2 (Treestar) software.

### In vivo B2m^-/-^ rejection

Mice were treated with tamoxifen in corn oil by oral gavage for 2 or 3 consecutive days. On the day after the last dose of tamoxifen, mice also received 2–4 x 10^6^ splenocytes that were a 1:1 mix of CD45.2 WT and B2m^-/-^ (RRID:IMSR_JAX:002087). Transferred splenocytes were differentially labelled with CellTrace^TM^ Violet Cell Proliferation Kit (CTV, Invitrogen C34557) for discrimination. Some mice received 200 µg anti-NK1.1 intraperitoneally to control for non-NK-specific killing the day before transfer. 18-20 h after splenocyte transfer, mice were sacrificed and the ratio of CTV^hi^ (WT) to CTV^low^ (B2m^-/-^) splenocytes was assessed. Percent rejection was calculated as: (1-[(Ratio B2m^-/-^: WT)_experimental_/(Ratio B2m^-/-^: WT)_NK depleted_])*100^45^.

### B16-F10 lung metastasis model

Ncr1-T-bet^Δ/Δ^ and T-bet WT mice were treated with 1 mg tamoxifen by oral gavage for 3 days, on Day 4, 2.5 x 10^5^ B16-F10 cells were injected i.v.. Eleven days later, lungs were harvested, and lung nodules were counted by a blinded counter.

### In vitro functional assays

Splenocytes and processed liver cells were either unstimulated (media with low dose IL-15) or stimulated with additional cytokines (10 ng/mL IL-12 (Peprotech #210-12), additional 10 ng/mL IL-15 (Peprotech #210-15), and/or 10 ng/mL IL-18 (Gibco PMC0184), YAC-1 lymphoma targets (E:T = 10:1), or 2.5 μg plate-bound purified anti-NK1.1 for 6 h. Anti-CD107a (LAMP-1) antibody (Invitrogen 50-1071-82) was added at the start of the assay, and brefeldin A (BD BDB555029) and monensin (BD BDB554724) were added after the first hour. Cells were then stained for surface antigens, fixed/permeabilized using the BD Biosciences Fix/Perm kit, intracellularly stained for IFN-γ, and analyzed by flow cytometry.

### In-vitro cytotoxicity (FloKA) assay

Splenocytes were plated at 1.0 x 10^7^/mL in a 12-well plate in RPMI-1640 plus 10% FBS, supplements, and 50 ng/mL IL-15 for 48 h at 37 °C incubator. Stimulated splenocytes were used in killing assays against CTV-labeled RMA-S targets at the indicated E:T ratios for 4-6 h. Dead cells were identified via 7-AAD (Biolegend 420404) staining and specific killing calculated as: % 7-AAD^+^_effector+target_ - % % 7-AAD^+^_target alone_.

### Phospho-STAT4 assessment

Splenocytes were plated at 1e6 per well in a 96-well plate in RPMI-1640 plus 10% FBS and supplements and let rest for 30 minutes to 1 hour at a 37 °C incubator. After resting, the splenocytes were stimulated with 10 ng/mL IL-12 for 1 h. At the end of the incubation, 4% PFA was immediately added to the wells to a final working concentration of 1% for 10 minutes at room temperature. After centrifuging and removing the supernatant, the cells were then incubated in ice-cold methanol for 30 min at 4 °C before staining with pSTAT4 (pY693) antibody (BD 558137).

### RNA-sequencing

T-bet WT mice and T-bet Δ/Δ mice were treated with 1 mg tamoxifen by oral gavage for 2 days (Day 1-3), then on Day 3, liver NK cells (YFP^+^NK1.1^+^CD49a^-^CD49b^+^) and ILC1 (YFP^+^NK1.1^+^CD49a^+^CD49b^-^) from the same mice were flow sorted. Qiagen RNeasy micro kit (74004) was used to extract RNA of cells directly sorted into Buffer RLT + β-mercaptoethanol, following the manufacturer’s protocol. The sequencing library was prepared using the SMARTer Ultra Low RNA kit for Illumina Sequencing (Takara-Clontech) and sequenced on Illumina NovaSeq X Plus. R/Bioconductor package Limma was used to perform differential expression analysis.

### MCMV infection

Age and sex-matched mice were infected with 2 ×105 plaque forming units (pfu) i.p. of Smith strain MCMV for a survival study. Sub-lethal MCMV challenge was performed by infecting mice with 5 ×10^4^ pfu i.p. and euthanzing the animals at the indicated time points post infection for viral titers by qPCR and flow cytometric assessment as previously described^46^.

### Quantification and statistical analysis

Statistical analyses were performed using GraphPad Prism (v10.6.1) software. Tests utilized are indicated in the figure legends. Summary data are shown as mean ± SEM; * = *p* < 0.05, ** = *p* < 0.01, *** = *p* < 0.001, **** = *p* < 0.0001.

### Materials availability

The tamoxifen inducible Ncr1-T-bet^Δ/Δ^, Ncr1-T-bet^Δ/Δ^Eomes^Δ/Δ^ mice generated in this study will be made available to qualified investigators by the corresponding author, Todd A. Fehniger (tfehnige@wustl.edu) upon request.

### Reporting summary

Further information on research design is available in the Nature Portfolio Reporting Summary linked to this article.

## Supplementary information

Supplementary Information

Reporting Summary

Transparent Peer Review file

## Source data

Source data

## Data Availability

The RNaseq data generated in this study have been deposited in the Gene Expression Omnibus data base under accession code GSE309461. The raw numbers for figures are available in the Source Data file. Source data are provided with this paper.
