# Supplementary material for: Inducible T-bet deletion reveals tissue-specific requirements in NKp46+ ILC immunobiology and response to murine cytomegalovirus
Source: Nat Commun. 2026 Jun 18;17:7708. doi: 10.1038/s41467-026-74478-y (PMC13434699; doi:10.1038/s41467-026-74478-y)
Supplement: Supplementary file 1 [file 41467_2026_74478_MOESM1_ESM.pdf]

Supplementary Information

Inducible T-bet deletion reveals tissue-specific requirements in NKp46$^+$ ILC immunobiology and response to murine cytomegalovirus

Pamela Wong[1*], Yeeun Paik[1*], Lily Chang[1], Allison Burdi[1], Carly Neal[1], Celia C. Cubitt[1], Jennifer Tran[1], Bishan Bhattarai[2], Lyra Morina[1] , Nancy D. Marin[1], Kimberly Hwang[1], Hyogon Sohn[3], Julia A. Wagner[1], Jennifer A. Foltz[1], Wilbur M. Song[1], Timothy Schappe[1], Lynne Marsala[1], Mark Foster[1], Julia Foster[1], Melissa M. Berrien-Elliott[1], Sytse J Piersma[4], Marco Colonna[2], Wayne M. Yokoyama[4], Megan A. Cooper[3], Todd A. Fehniger[1#]

[1]Division of Oncology, Department of Medicine, Washington University School of Medicine, St. Louis, MO 63110 USA

[2]Department of Pathology and Immunology, Washington University School of Medicine, St. Louis, MO 63110 USA

[3]Division of Rheumatology and Immunology, Department of Pediatrics, Washington University School of Medicine, St. Louis, MO 63110 USA

[4]Division of Rheumatology, Department of Medicine, Washington University School of Medicine, St. Louis, MO 63110 USA

*These authors contributed equally.

[#]Correspondence: tfehnige@wustl.edu

Table of Contents

Suppl. Fig. 1: Supporting data for figure 1 and Figure 2a-b

Suppl. Fig. 2: Supporting data for figure 1

Suppl. Fig. 3: Sorted Stage II/III splenic NK cells do not mature to Stage IV *in vitro.*

Suppl. Fig. 4: Most splenic NK1.1^{+}NKp46^{+} cells are CD49b^{+} CD49a^{-}.

Suppl. Fig. 5: Supporting data for figure 3

Suppl. Fig. 6: Supporting data for figure 4

Suppl. Fig. 7: Supporting data for figure 3 and figure 4

Suppl. Fig. 8: Supporting data for figure 5

Suppl. Fig. 9: Supporting data for figure 6

Suppl. Table 1: Antibodies

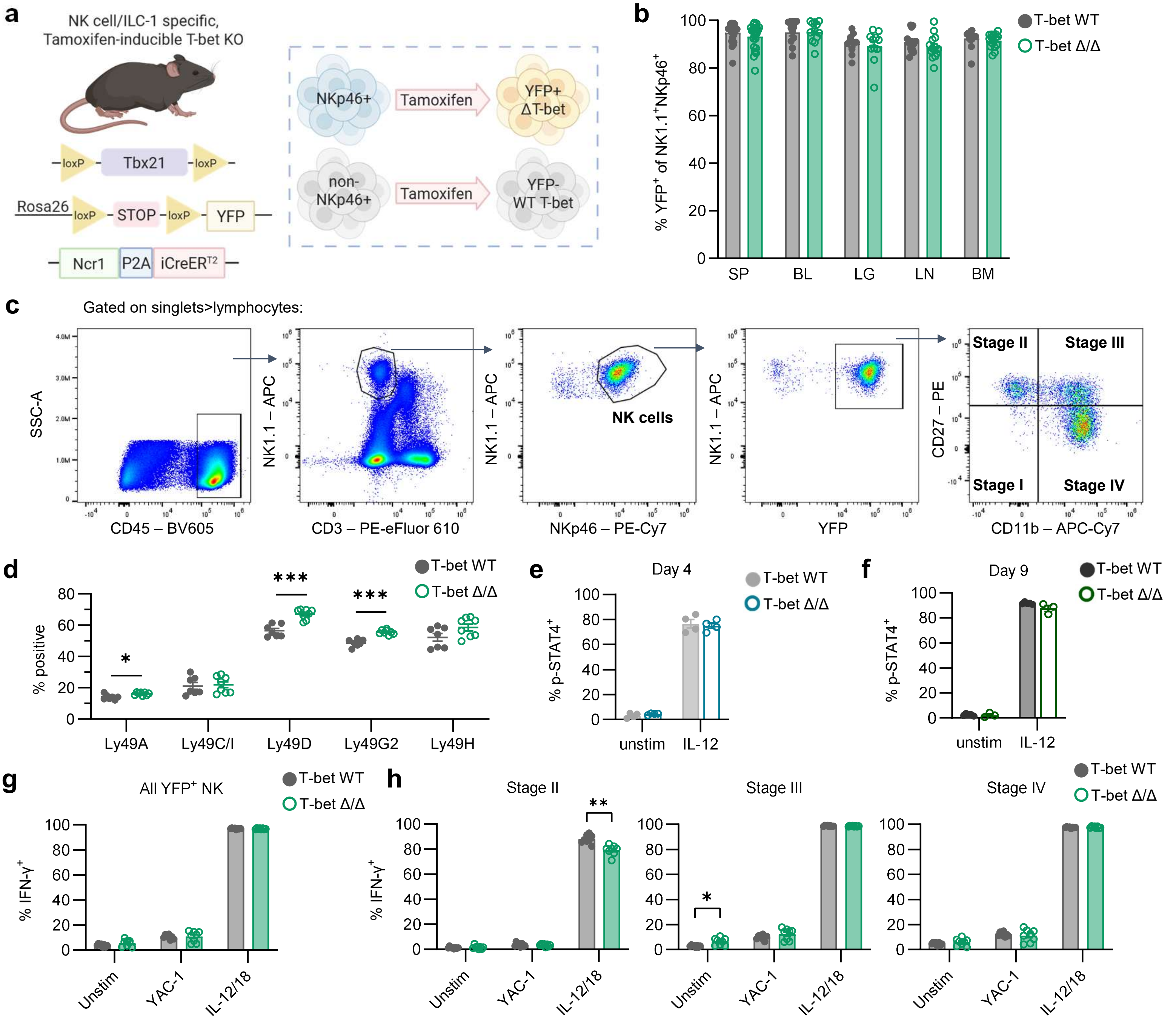

Supplementary Figure 1. *Ncr1*-iCreER^{T2} x *Rosa26*-YFP^{LSL} x Tbx21$^{fl/fl}$ mouse. *Ncr1*-iCreER^{T2} x *Rosa26*-YFP^{LSL} x *Tbx21*$^{fl/fl}$ (T-bet Δ/Δ) mice and Ncr1-iCreER^{T2} x Rosa26-YFP^{LSL} x Tbx21$^{WT/WT}$ (T-bet WT) mice were treated with 1 mg tamoxifen by oral gavage for 3 days (Day 1-3), then on Day 6, splenocytes were harvested for ex vivo phenotypic and functional assessment. **a**, Schema of Ncr1-Tbx21$^{Δ/Δ}$ mouse model. Created in BioRender. Fehniger, T. (2026) https://BioRender.com/dtwdpmg. **b**, YFP$^+$ percentage of NK1.1$^+$NKp46$^+$ NK cells in indicated tissues (n=10-24 per group). **c**, Representative flow gating strategy. **d**, Ly49 expression on YFP$^+$ splenic NK cells (n=7-8 per group). Phospho-STAT4 percentage of splenic NK cells from T-bet WT and T-bet Δ/Δ at **e**, Tam-4d (n=4 per group) and **f**, Tam-9d (n=3-5 per group) after stimulation with IL-12. IFN-γ production by **g**, all YFP$^+$ NK cells (n=8 per group) and **h**, YFP$^+$ NK cells within indicated maturation stage (n=8 per group) in response to IL-12+18 and YAC-1 stimulation at tam-6d. Summary data shown as mean ± SEM; Statistical significance determined by b and d, multiple unpaired t-test and e-h, 2-way ANOVA with Šídák's multiple comparisons test. 2-3 independent experiments. Exact p-values are provided in the Source Data file.

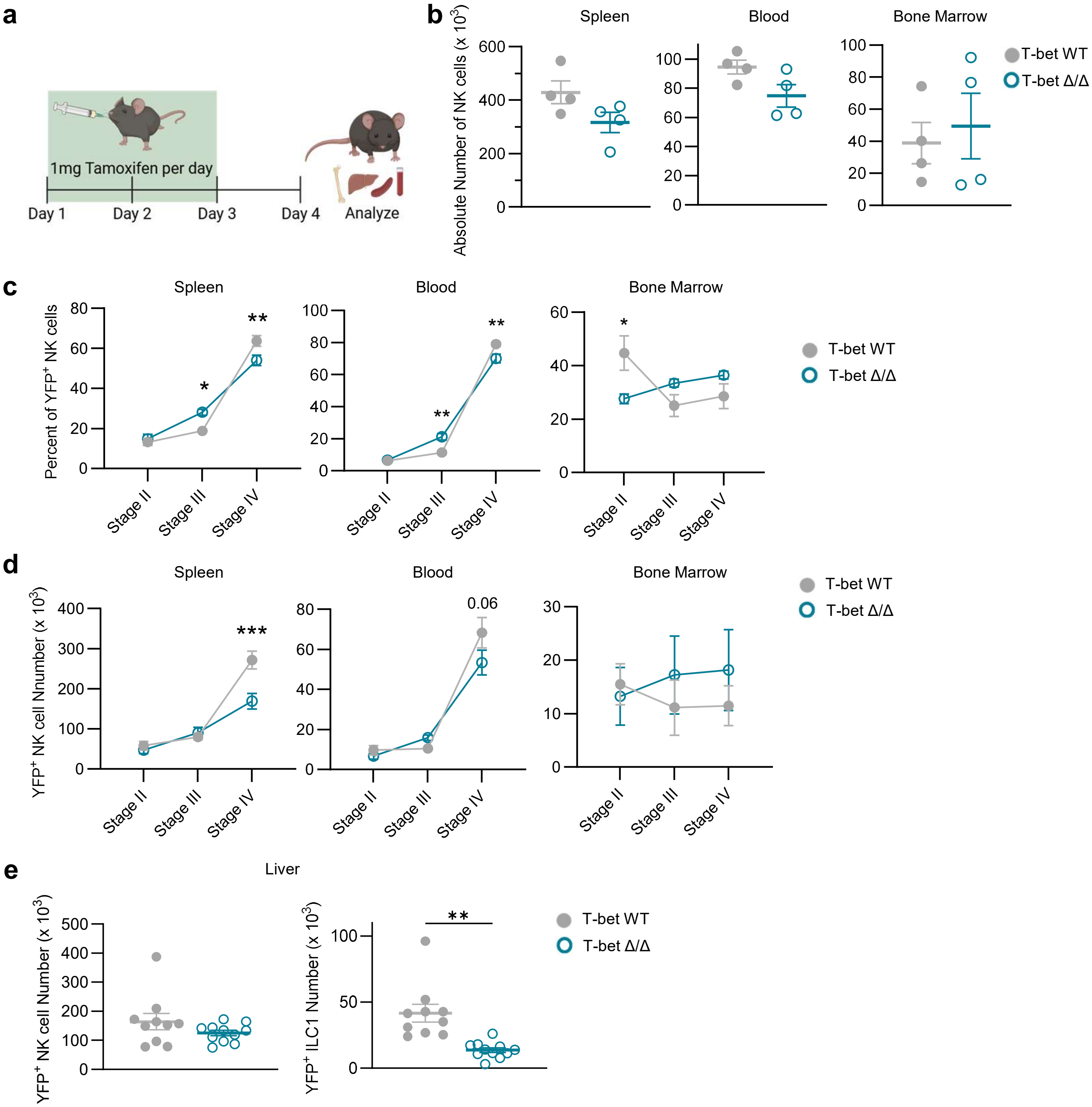

Supplementary Figure 2. Loss of Stage IV NK cells and ILC1s are observed as early as day 4 of tamoxifen regiment. a, *Ncr1*-iCreER^{T2} x *Rosa26*-YFP^{LSL} x *Tbx21*$^{fl/fl}$ (T-bet Δ/Δ) mice and Ncr1-iCreER^{T2} x Rosa26-YFP^{LSL} x Tbx21$^{WT/WT}$ (T-bet WT) mice were treated with 1 mg tamoxifen by oral gavage for 3 days (Day 1-3), then on Day 4, mice were sacrificed for analysis. Created in BioRender. Fehniger, T. (2026) https://BioRender.com/kd95jaw. **b**, YFP^{+} NK cell numbers (CD3^{-}NK1.1^{+}NKp46^{+}) in indicated organs (n=4 per group). **c**, percentage (n=4 per group) and **d**, absolute numbers (n=4 per group) of YFP^{+} NK cell within each indicated maturation stage. **e**, Absolute numbers of YFP^{+} NK cell (CD3^{-}NK1.1^{+}NKp46^{+}CD49a^{-}CD49b^{+}) and ILC1 (CD3^{-}NK1.1^{+}NKp46^{+}CD49a^{+}CD49b^{-}) in liver (n=10-11 per group). Summary data shown as mean ± SEM; Statistical significance was determined by b and e, unpaired two-sided t-test with Welch's correction, and c-d, 2-way ANOVA with Šídák's multiple comparisons test. 2-4 independent experiments. Exact p-values are provided in the Source Data file.

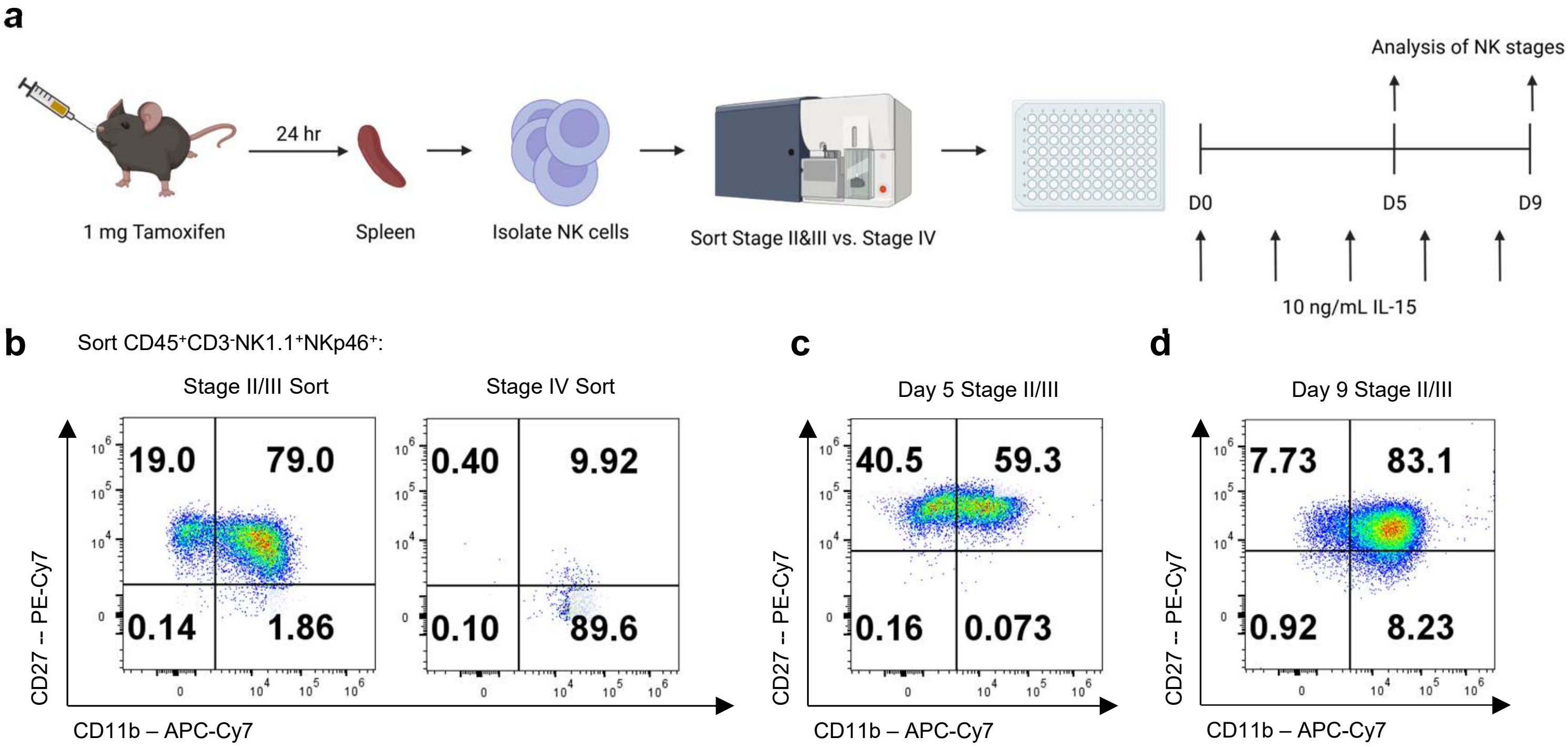

Supplementary Figure 3. Sorted Stage II/III splenic NK cells do not mature to Stage IV *in vitro.* iCreER^{T2} x Rosa26-YFP^{LSL} control mice were treated with 1 mg tamoxifen by oral gavage, then 24 hours later, splenic NK cells ($CD45^+CD3^-NK1.1^+NKp46^+$) were isolated and stage II/III ($CD27^+$) NK cells were sorted and plated with 10 ng/mL IL-15 to induce in vitro differentiation. **a**, Schema. Created in BioRender. Fehniger, T. (2026) https://BioRender.com/zufe36a. **b**, post-sort analysis of Stage II/III vs. IV NK cells ($CD45^+CD3^-NK1.1^+NKp46^+$). **c-d**, NK stage analysis c, 5-days post sort and d, 9-days post sort.

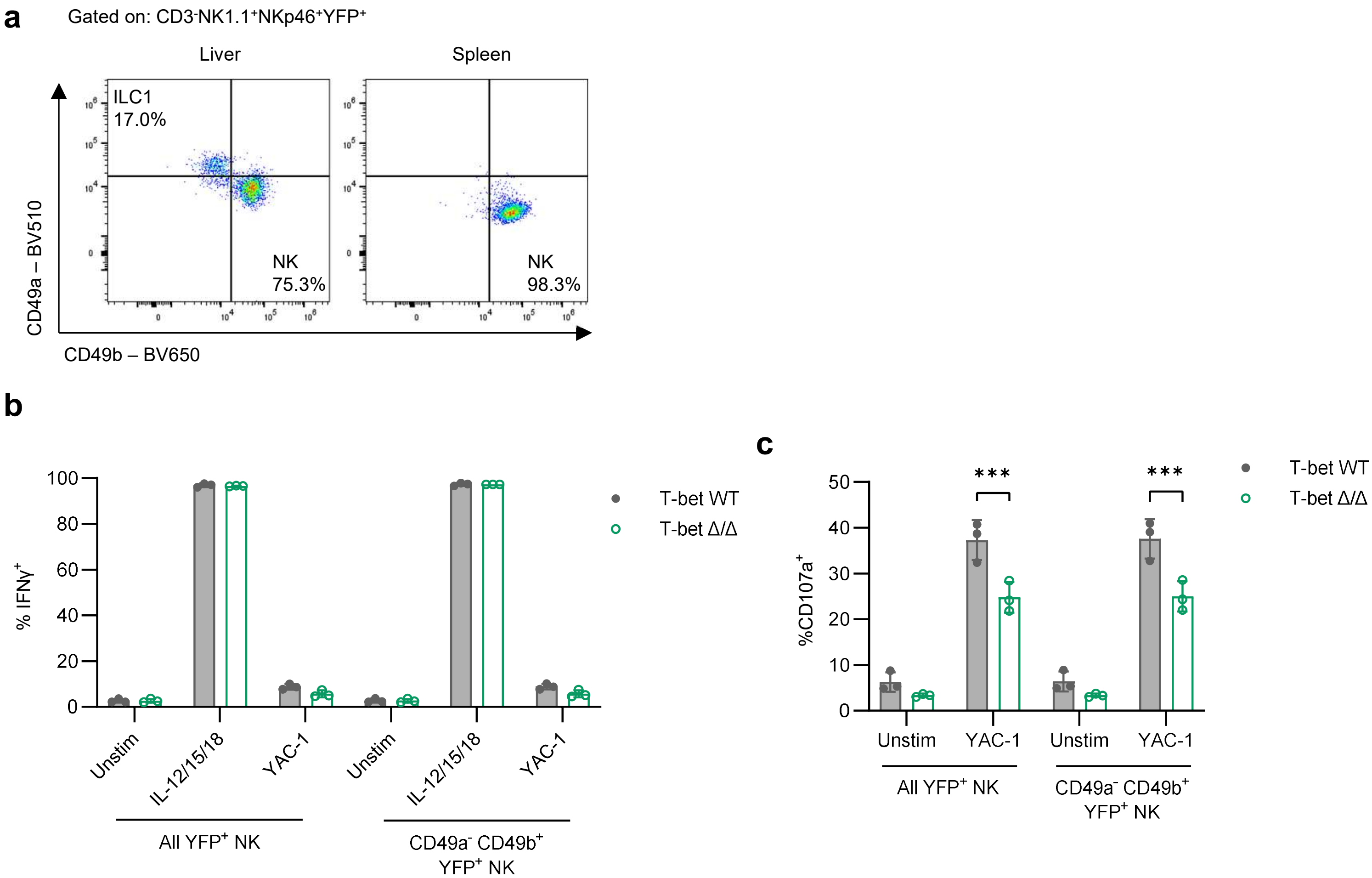

Supplementary Figure 4. Gating on NK1.1⁺NKp46⁺ NK cells that are CD49b⁺CD49a⁻ does not impact functional responses of T-bet Δ/Δ NK cells. *Ncr1*-iCreER^{T2} x *Rosa26*-YFP^{LSL} x *Tbx21*$^{fl/fl}$ (T-bet Δ/Δ) mice and Ncr1-iCreER^{T2} x Rosa26-YFP^{LSL} x Tbx21$^{WT/WT}$ (T-bet WT) mice were treated with 1 mg tamoxifen by oral gavage for 3 days (Day 1-3), then on Day 6, splenocytes were harvested for ex vivo functional assessment. **a**, Representative flow plot of CD3$^-$NK1.1$^+$NKp46$^+$ from liver vs. spleen. **b-c** side-by-side comparison of b, IFN-γ production (n=3 per group) and c, CD107a$^+$ population (n=3 per group) within splenic NK calculated using the manuscript's gating strategy (left) vs. additional gating on CD49b$^+$ CD49a$^-$ population (right). Only male mice were used in this figure. Statistical significance was determined by 2-way ANOVA with Šídák's multiple comparisons test. Exact p-values are provided in the Source Data file.

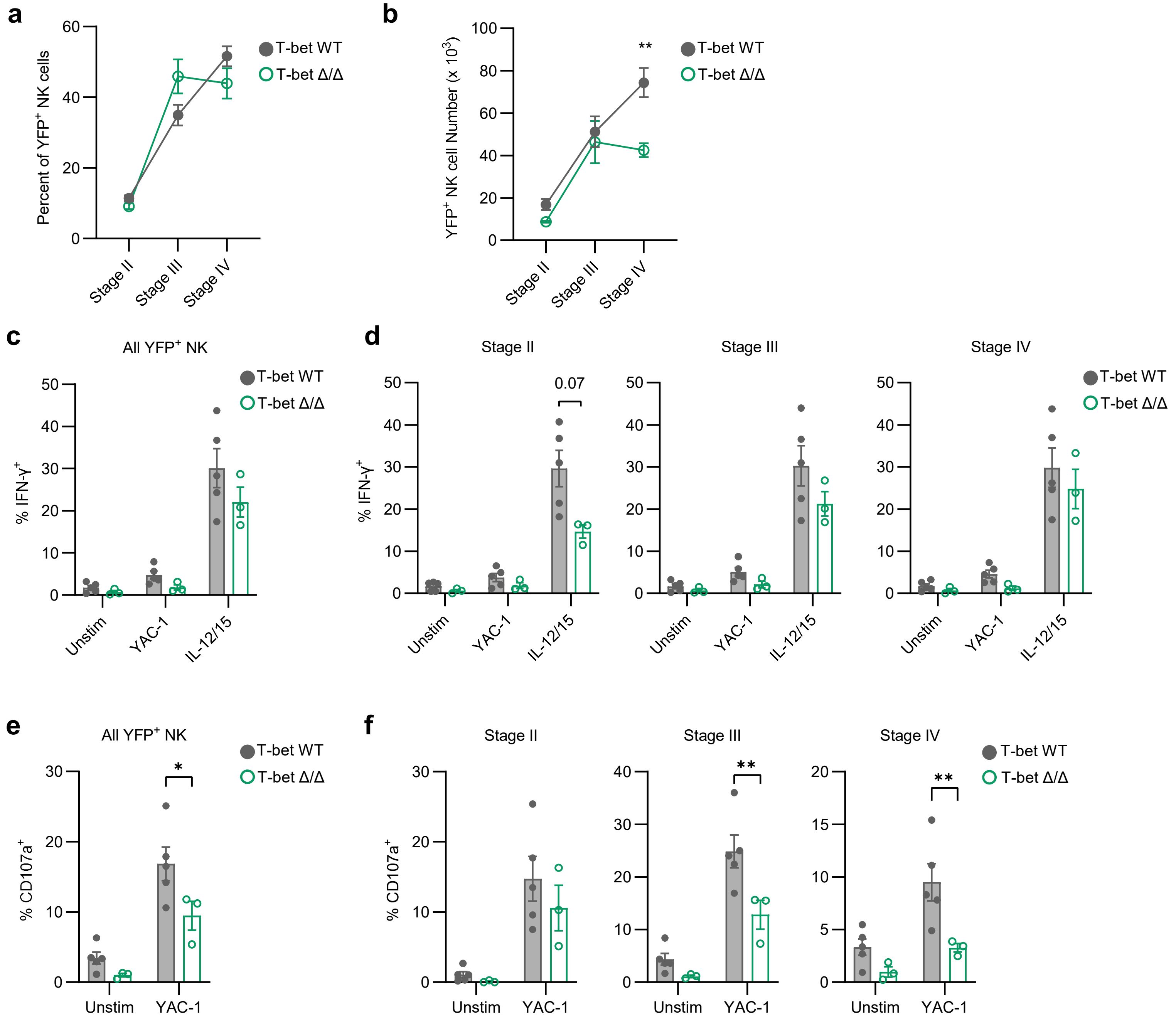

Supplementary Figure 5. IFN-γ response is preserved but degranulation is impaired in Ncr1-T-bet$^{Δ/Δ}$ Liver NK cells. *Ncr1*-iCreER^{T2} x *Rosa26*-YFP^{LSL} x *Tbx21*$^{fl/fl}$ (T-bet Δ/Δ) mice and Ncr1-iCreER^{T2} x Rosa26-YFP^{LSL} x Tbx21$^{WT/WT}$ (T-bet WT) mice were treated with 1 mg tamoxifen by oral gavage for 3 days (Day 1-3), then on Day 6, Liver cells were harvested. **a-b** Summary data of a, percentage (n=3-4 per group) and b, absolute numbers (n=3-4 per group) of YFP^{+} NK cells within each indicated stage. **c-f** ex-vivo assessment of IFN-γ production (n=3-5 per group) and degranulation (n=3-5 per group) in response to YAC-1 stimulation by c, all YFP^{+} NK cells (CD3^{-} NK1.1^{+}NKp46^{+}CD49a^{-}CD49b^{+}) and d, YFP^{+} NK cells within indicated maturation stage in response to YAC-1 and IL12+15. CD107a^{+} percentage within e, YFP^{+} NK cells and f, YFP^{+} NK cells within indicated maturation stage in response to YAC-1 stimulation. Only male mice were used in this figure. Summary data shown as mean ± SEM; Statistical significance was determined by 2-way ANOVA with Šídák's multiple comparisons test. 2 independent experiments. Exact p-values are provided in the Source Data file.

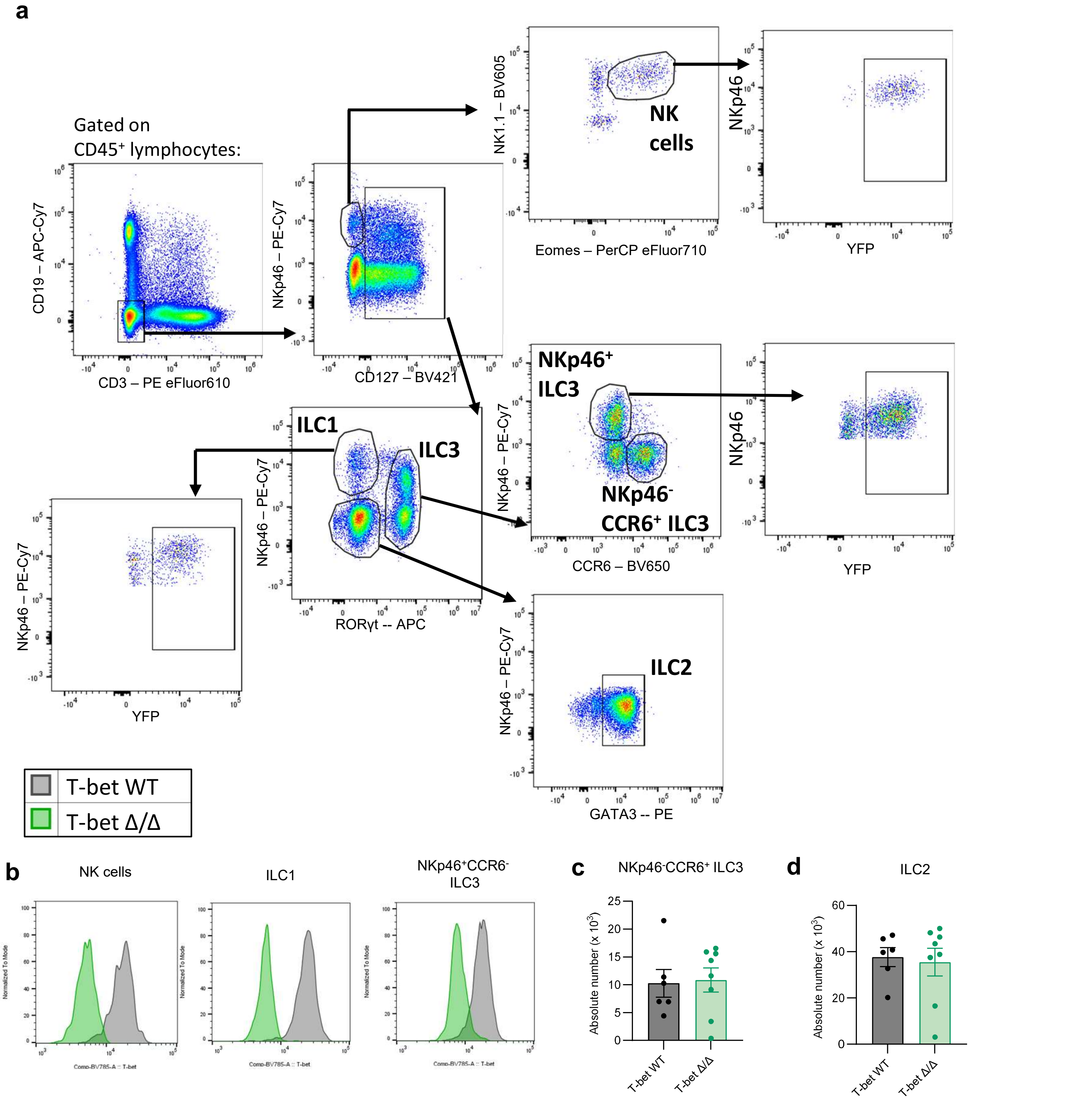

Supplementary Figure 6. NKp46$^-$CCR6$^+$ ILC3 and ILC2 numbers in the lamina propria were not impacted by inducible NKp46$^+$ cell-specific T-bet deletion. *Ncr1*-iCreER^{T2} x *Rosa26*-YFP^{LSL} x *Tbx21*$^{fl/fl}$ (T-bet Δ/Δ) mice and Ncr1-iCreER^{T2} x Rosa26-YFP^{LSL} x Tbx21$^{WT/WT}$ (T-bet WT) mice were treated with 1 mg tamoxifen by oral gavage for 3 days (Day 1-3), then on Day 6, mice were harvested for analysis. **a**, Flow gating scheme of lamina propria innate lymphoid cell subsets. Summary data of absolute number of **b**, Representative flow plots of T-bet expression of YFP$^+$ cells of indicated innate lymphoid cell subset on Day 6 of tamoxifen regiment. Absolute Number of YFP$^+$ **c**, NKp46$^-$CCR6$^+$ ILC3 (n=6-8 per group) and **d**, ILC2 (n=6-8 per group) on Day 6 of tamoxifen regiment. Summary data shown as mean ± SEM; Statistical significance calculated by unpaired two-tailed t-test with Welch's correction. N= 6-8 from 3 independent experiments. Exact p-values are provided in the Source Data file.

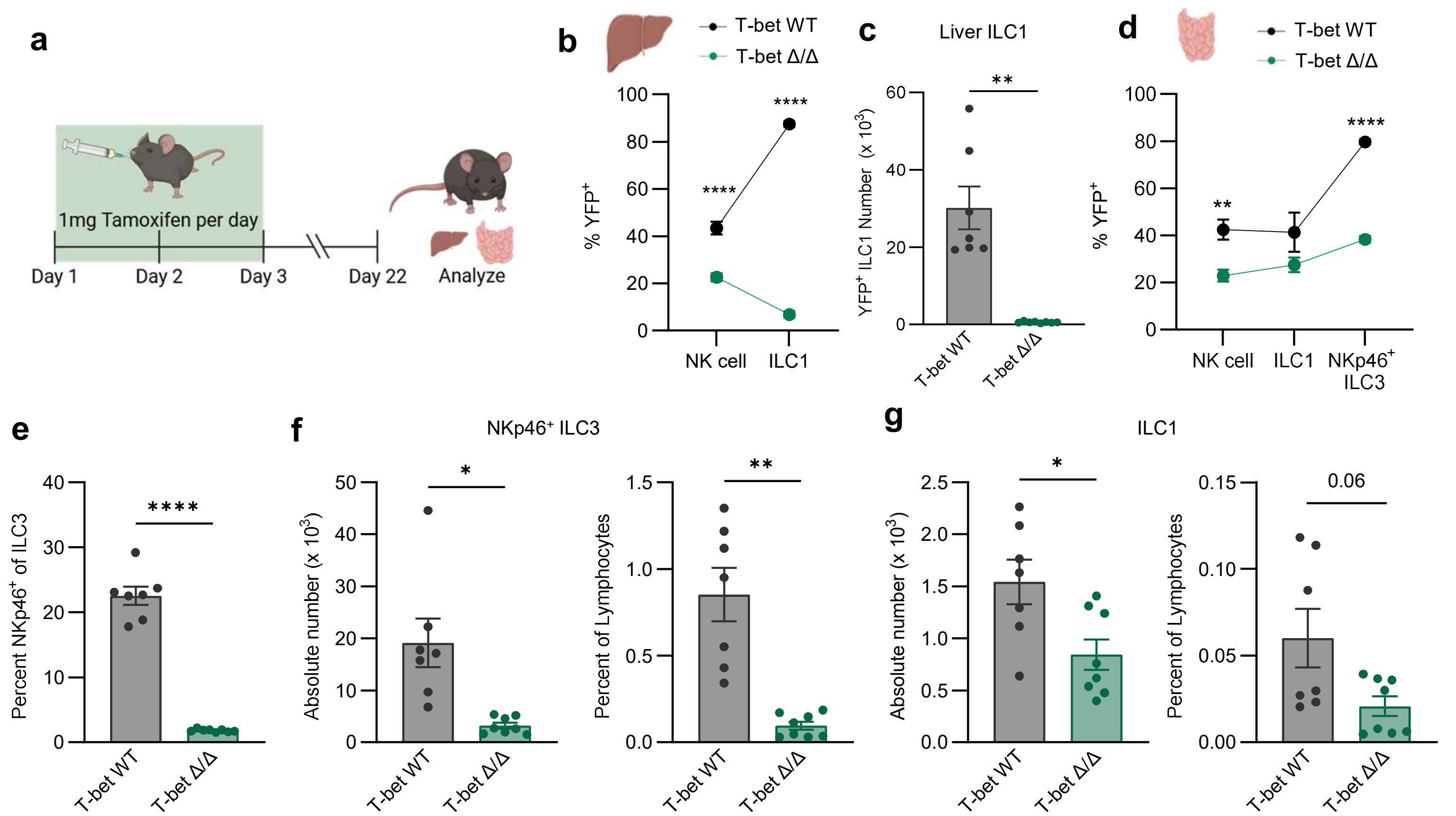

Supplementary Figure 7. Liver ILC1s, small intestine lamina propria NKp46+ ILC3s, and ILC1s require sustained expression of T-bet for long-term persistence. a, *Ncr1*-iCreER^{T2} x *Rosa26*-YFP^{LSL} x *Tbx21*$^{fl/fl}$ (T-bet Δ/Δ) mice and Ncr1-iCreER^{T2} x Rosa26-YFP^{LSL} x Tbx21$^{WT/WT}$ (T-bet WT) mice were treated with 1 mg tamoxifen by oral gavage for 3 days (Day 1-3), then on Day 22, mice were analyzed. Created in BioRender. Fehniger, T. (2026) https://BioRender.com/ho7fm50. **b**, Liver YFP+ percentage of indicated subset (n=7-8 per group) and **c,** absolute number of YFP+ ILC1 (n=7-8 per group). **d**, Small Intestine lamina propria YFP+ percentage of indicated subset (n=7-8 per group). **e**, Summary data of YFP+ NKp46+ ILC3s in the small intestine lamina propria of T-bet WT and T-bet FKO mice (n=7-8 per group). **f-g**, YFP+ NKp46+ ILC3s (n=7-8 per group) and YFP+ ILC1s (n=7-8 per group) as absolute numbers and percentage of lymphocytes in the small intestine lamina propria. Summary data shown as mean ± SEM; Statistical significance determined by b and d, 2-way ANOVA with Šídák's multiple comparisons test for adjusted p-values and c and e-g, unpaired two-tailed t-test with Welch's correction. Two independent experiments. Exact p-values are provided in the Source Data file.

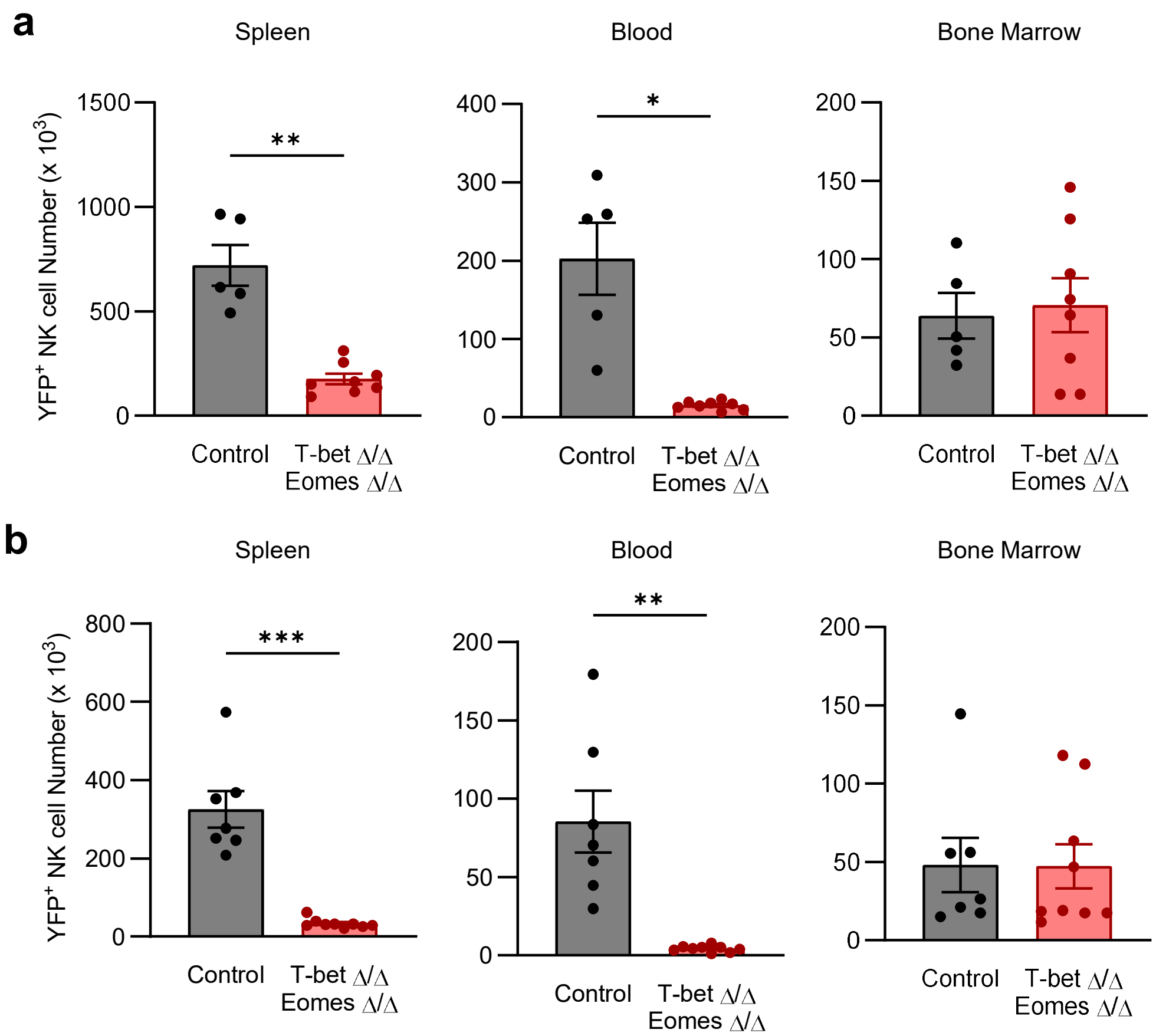

Supplementary Figure 8. Inducible deletion of both T-bet and Eomes led to prolonged loss of NK cells in the spleen and blood but not in the bone marrow. *Ncr1*-iCreER^{T2} x *Rosa26*-YFP^{LSL} x *Tbx21*$^{fl/fl}$ x *Eomes*$^{Δ/Δ}$ (T-bet Δ/Δ Eomes Δ/Δ) or *Ncr1*-iCreER^{T2} x *Rosa26*-YFP^{LSL} (control) mice were treated with 1 mg per day tamoxifen by oral gavage for 3 days (Day 1-3), then on **a,** day 6 (n=5-8 per group) and **b,** day 9 (n=7-9 per group) absolute numbers of YFP$^+$ NK cells within each indicated organs were analyzed. Summary data shown as mean ± SEM; Statistical significance determined with unpaired two-tailed t-test with Welch's correction. Three independent experiments. Exact p-values are provided in the Source Data file.

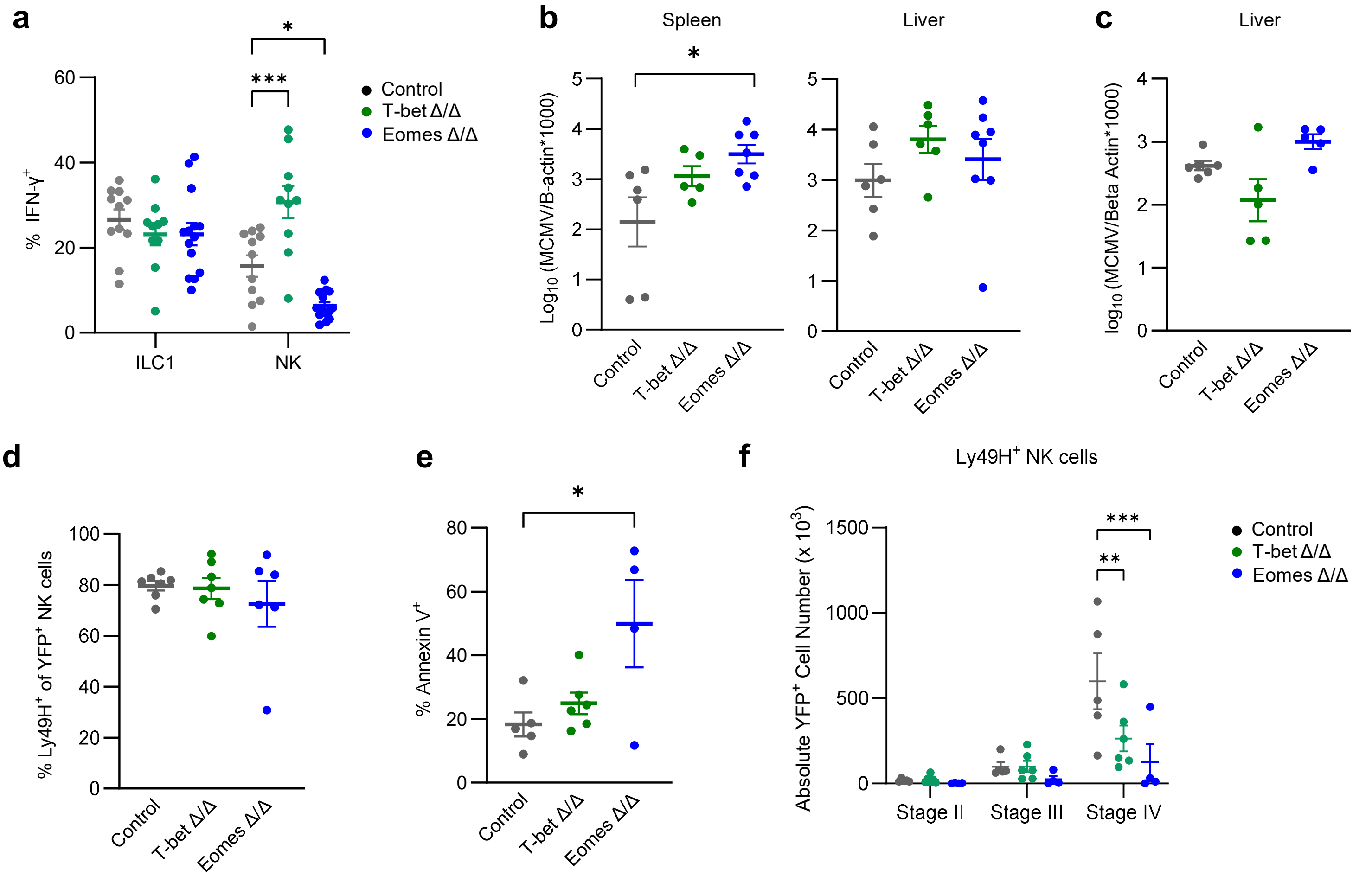

Supplementary Figure 9. T-bet is dispensable while Eomes is required for protective MCMV response. *Ncr1*-iCreER^{T2} x *Rosa26*-YFP^{LSL} x *Tbx21*$^{fl/fl}$ (T-bet Δ/Δ), *Ncr1*-iCreER^{T2} x *Rosa26*-YFP^{LSL} x *Eomes*$^{fl/fl}$ (Eomes Δ/Δ), and N*cr1*-iCreER^{T2} x *Rosa26*-YFP^{LSL} (control) mice were fed tamoxifen chow for 3 days. The day after tamoxifen chow was removed, the mice were infected with 5 x 10^4 pfu MCMV. **a**, IFN-γ$^+$ percentages within YFP$^+$ ILC1 population in the liver at 1.5 days post MCMV infection (n=10-14 per group). MCMV titer at **b**, 1.5 days (n=5-8 per group) and **c**, 4 days (n=5-6 per group) post infection in indicated tissue. **d**, Ly49H$^+$ KLRG1$^+$ percentage within YFP$^+$ NK cells on 7 days post infection (n=7-8 per group). **e**, Ex vivo assessment of Annexin V expression within YFP$^+$ NK cells 7 days post infection (n=4-6 per group). **f**, Absolute YFP$^+$ Ly49H$^+$ NK cell numbers within indicated maturation stages in the spleen 7 days post infection (N=4-6 per group). Summary data shown as mean ± SEM; Statistical significance was determined by two way ANOVA with Šidák's multiple comparison test in a and f, and one way ANOVA with Dunnett's multiple-comparisons test in b-e. 2-4 independent experiments. Exact p-values are provided in the Source Data file.

Target	Fluorophore	Titer	Brand	Catalog	Clone
CD3e	PE-eFluor610	1:100	Invitrogen	61-0031-82	145-2C11
	Pacific Blue	1:100	BD	BDB558214	500A2
CD11b	APC-Cy7	1:100	BD	BDB557657	M1/70
CD19	APC-Cy7	1:100	Biolegend	115530	6D5
CD27	PE	1:100	Biolegend	124209	LG.3A10
	PE-Cy7	1:500	Biolegend	25-0271-82	LG.7F9
CD45	BV510	1:50	Biolegend	103137	30-F11
	BV605	1:100	Biolegend	103140	30-F11
	PerCP-Cy5.5	1:100	BD	BDB550994	30-F11
CD49a	BV510	1:100	BD	BDB740144	Ha31/8
	PE	1:100	BD	BDB562115	Ha31/8
CD49b	BV650	1:100	BD	BDB740496	HMα2
	APC	1:50	Invitrogen	17-5971-82	DX5
CD62L	APC-Cy7	1:200	Biolegend	104427	MEL-14
CD107a	eFluor660	1:200	Invitrogen	50-1071-82	1D4B
CD122	BV711	1:20	BD	BDB740679	TM-B1
CD127	BV421	1:50	Biolegend	135023	A7R34
Ly49A	PE	1:20	Biolegend	138703	A1
Ly49C/I	BV510	1:20	BD	BDB744028	5E6
Ly49D	BV650	1:100	BD	BDB742558	4E5
Ly49G2	PerCP-eFluor710	1:50	Invitrogen	46-5781-82	4D11
Ly49H	APC	1:100	Invitrogen	17-5886-82	3D10
NK1.1	BV605	1:100	Biolegend	108740	PK136
	PerCP-Cy5.5	1:50	BD	BDB551114	PK136
	Alexa Fluor 700	1:50	Invitrogen	56-5941-82	PK136
	APC	1:100	BD	BDB550627	PK136
NKp46	eFluor450	1:20	Invitrogen	48-3351-82	29A1.4
	PE	1:50	Invitrogen	12-3351-82	29A1.4
	PE-Cy7	1:50	Invitrogen	25-3351-82	29A1.4
KLRG1	BV785	1:50	Biolegend	138429	MAFA
	PE-Cy7	1:100	Invitrogen	25-5893-82	2F1
IFN-γ	PE	1:50	Invitrogen	12-7311-82	XMG1.2
	BV750	1:50	Biolegend	505865	XMG1.2
Granzyme B	PE Cy5.5	1:100	Invitrogen	GRB18	GB11
	APC	1:50	Invitrogen	MHGB05	GB11
T-bet	BV421	1:20	Biolegend	644816	4B10
	BV785	1:20	Biolegend	644835	4B10
EOMES	PerCP-eFluor710	1:400	Invitrogen	46-4875-82	Dan11mag
GATA3	PE	1:100	BD	BDB560074	L50-823
CCR6	BV650	1:50	BD	BDB740441	140706
RORγT	APC	1:100	Invitrogen	17-6988-82	AFKJS-9
pSTAT4	Alexa Fluor 647	1:20	BD	BDB558137	pY693
Annexin V	PE	1:20	BD	BDB556422	
	APC	1:100	BD	BDB550474	
7-AAD		1:250	Biolegend	420404	
GhostDyeRed 710		1:4000	Cytek	13-0871-T500	
Far-Red Fluorescent FLICA® 660 Caspase-3/7 (DEVD) Assay Kit	FLICA 660	1:50	Immunochemistry technologies	9125	

Supplementary Table 1. Antibodies
