# Supplementary material for: Inducible T-bet deletion reveals tissue-specific requirements in NKp46+ ILC immunobiology and response to murine cytomegalovirus
Source: Nat Commun. 2026 Jun 18;17:7708. doi: 10.1038/s41467-026-74478-y (PMC13434699; doi:10.1038/s41467-026-74478-y)
Supplement: Supplementary file 2 [file 41467_2026_74478_MOESM2_ESM.pdf]

nature portfolio

Corresponding author(s): todd a fehniger

Last updated by author(s): 02-28-2026

Reporting Summary

Nature Portfolio wishes to improve the reproducibility of the work that we publish. This form provides structure for consistency and transparency in reporting. For further information on Nature Portfolio policies, see our Editorial Policies and the Editorial Policy Checklist.

Statistics

For all statistical analyses, confirm that the following items are present in the figure legend, table legend, main text, or Methods section.

n/a	Confirmed	
☐	☒	The exact sample size (*n*) for each experimental group/condition, given as a discrete number and unit of measurement
☐	☒	A statement on whether measurements were taken from distinct samples or whether the same sample was measured repeatedly
☐	☒	The statistical test(s) used AND whether they are one- or two-sided<br>*Only common tests should be described solely by name; describe more complex techniques in the Methods section.*
☐	☒	A description of all covariates tested
☐	☒	A description of any assumptions or corrections, such as tests of normality and adjustment for multiple comparisons
☐	☒	A full description of the statistical parameters including central tendency (e.g. means) or other basic estimates (e.g. regression coefficient) AND variation (e.g. standard deviation) or associated estimates of uncertainty (e.g. confidence intervals)
☐	☒	For null hypothesis testing, the test statistic (e.g. F, t, r) with confidence intervals, effect sizes, degrees of freedom and P value noted<br>*Give P values as exact values whenever suitable.*
☒	☐	For Bayesian analysis, information on the choice of priors and Markov chain Monte Carlo settings
☒	☐	For hierarchical and complex designs, identification of the appropriate level for tests and full reporting of outcomes
☒	☐	Estimates of effect sizes (e.g. Cohen's *d*, Pearson's *r*), indicating how they were calculated

Our web collection on statistics for biologists contains articles on many of the points above.

Software and code

Policy information about availability of computer code

Data collection	N/A
Data analysis	N/A

For manuscripts utilizing custom algorithms or software that are central to the research but not yet described in published literature, software must be made available to editors and reviewers. We strongly encourage code deposition in a community repository (e.g. GitHub). See the Nature Portfolio guidelines for submitting code & software for further information.

Data

Policy information about availability of data

All manuscripts must include a data availability statement. This statement should provide the following information, where applicable:

- Accession codes, unique identifiers, or web links for publicly available datasets
- A description of any restrictions on data availability
- For clinical datasets or third party data, please ensure that the statement adheres to our policy

All primary data is available upon request by contacting the corresponding author. The RNA sequencing data is available at the Gene Expression Omnibus (GEO) with accession GSE309461.

Research involving human participants, their data, or biological material

Policy information about studies with human participants or human data. See also policy information about sex, gender (identity/presentation), and sexual orientation and race, ethnicity and racism.

Reporting on sex and gender	N/A
Reporting on race, ethnicity, or other socially relevant groupings	N/A
Population characteristics	N/A
Recruitment	N/A
Ethics oversight	N/A

Note that full information on the approval of the study protocol must also be provided in the manuscript.

Field-specific reporting

Please select the one below that is the best fit for your research. If you are not sure, read the appropriate sections before making your selection.

[x] Life sciences [ ] Behavioural & social sciences [ ] Ecological, evolutionary & environmental sciences

For a reference copy of the document with all sections, see nature.com/documents/nr-reporting-summary-flat.pdf

Life sciences study design

All studies must disclose on these points even when the disclosure is negative.

Sample size	Sample sizes were determined using prior studies of murine NK cells or MCMV as a guidance.
Data exclusions	No data was excluded.
Replication	Reproducibility was ensured by performing multiple independent experiments
Randomization	Randomization was not employed.
Blinding	Blinding was not employed.

Reporting for specific materials, systems and methods

We require information from authors about some types of materials, experimental systems and methods used in many studies. Here, indicate whether each material, system or method listed is relevant to your study. If you are not sure if a list item applies to your research, read the appropriate section before selecting a response.

Materials & experimental systems

n/a	Involved in the study	
[ ]	[x]	Antibodies
[ ]	[x]	Eukaryotic cell lines
[x]	[ ]	Palaeontology and archaeology
[ ]	[x]	Animals and other organisms
[x]	[ ]	Clinical data
[x]	[ ]	Dual use research of concern
[x]	[ ]	Plants

Methods

n/a	Involved in the study	
[x]	[ ]	ChIP-seq
[ ]	[x]	Flow cytometry
[x]	[ ]	MRI-based neuroimaging

Antibodies

Antibodies used

Listed below in order of target - fluorophore - titer - vendor - catalog # - Clone.

CD3e PE-eFluor610 1:100 Invitrogen 61-0031-82 145-2C11
CD3e Pacific Blue 1:100 BD BDB558214 500A2
CD11b APC-Cy7 1:100 BD BDB557657 M1/70
CD19 APC-Cy7 1:100 Biolegend 115530 6D5
CD27 PE 1:100 Biolegend 124209 LG.3A10

CD27 PE-Cy7 1:500 Biolegend 25-0271-82 LG.7F9
CD45 BV510 1:50 Biolegend 103137 30-F11
CD45 BV605 1:100 Biolegend 103140 30-F11
CD45 PerCP-Cy5.5 1:100 BD BDB550994 30-F11
CD49a BV510 1:100 BD BDB740144 Ha31/8
CD49a PE 1:100 BD BDB562115 Ha31/8
CD49b BV650 1:100 BD BDB740496 HMα2
CD49b APC 1:50 Invitrogen 17-5971-82 DX5
CD62L APC-Cy7 1:200 Biolegend 104427 MEL-14
CD107a eFluor660 1:200 Invitrogen 50-1071-82 1D4B
CD122 BV711 1:20 BD BDB740679 TM-B1
CD127 BV421 1:50 Biolegend 135023 A7R34
Ly49A PE 1:20 Biolegend 138703 A1
Ly49C/I BV510 1:20 BD BDB744028 5E6
Ly49D BV650 1:100 BD BDB742558 4E5
Ly49G2 PerCP-eFluor710 1:50 Invitrogen 46-5781-82 4D11
Ly49H APC 1:100 Invitrogen 17-5886-82 3D10
NK1.1 BV605 1:100 Biolegend 108740 PK136
NK1.1 PerCP-Cy5.5 1:50 BD BDB551114 PK136
NK1.1 Alexa Fluor 700 1:50 Invitrogen 56-5941-82 PK136
NK1.1 APC 1:100 BD BDB550627 PK136
NKp46 eFluor450 1:20 Invitrogen 48-3351-82 29A1.4
NKp46 PE 1:50 Invitrogen 12-3351-82 29A1.4
NKp46 PE-Cy7 1:50 Invitrogen 25-3351-82 29A1.4
KLRG1 BV785 1:50 Biolegend 138429 MAFA
KLRG1 PE-Cy7 1:100 Invitrogen 25-5893-82 2F1
IFN-γ BV750 1:50 Biolegend 505865 XMG1.2
IFN-γ PE 1:50 Invitrogen 12-7311-82 XMG1.2
Granzyme B PE Cy5.5 1:100 Invitrogen GRB18 GB11
Granzyme B APC 1:50 Invitrogen MHGB05 GB11
T-bet BV421 1:20 Biolegend 644816 4B10
T-bet BV785 1:20 Biolegend 644835 4B10
EOMES PerCP-eFluor710 1:400 Invitrogen 46-4875-82 Dan11mag
GATA3 PE 1:100 BD BDB560074 L50-823
CCR6 BV650 1:50 BD BDB740441 140706
RORγT APC 1:100 Invitrogen 17-6988-82 AFKJS-9
pSTAT4 Alexa Fluor 647 1:20 BD BDB558137 pY693
Annexin V PE 1:20 BD BDB556422
Annexin V APC 1:100 BD BDB550474

Viability staining:
7-AAD 1:250 Biolegend 420404
GhostDyeRed 710 1:4000 Cytek 13-0871-T500

FLICA Assay:
Far-Red Fluorescent FLICA® 660 Caspase-3/7 (DEVD) Assay Kit FLICA 660 1:50 Immunochemistry technologies 9125

Validation

All commercial antibodies were obtained from vendors that validated their specificity supplied as product information sheets.

Eukaryotic cell lines

Policy information about cell lines and Sex and Gender in Research

Cell line source(s)

YAC-1 (TIB-160), B16F10 (Male; CRL-6475) melanoma were obtained from ATCC. RMAS was a gift of Wayne Yokoyama.

Authentication

Cell lines were confirmed by phenotype at the time of receipt; cell lines were not authenticated by STR methods.

Mycoplasma contamination

All cell lines were tested for Mycoplasma using the MycoAlertPlus detection kit (Lonza) and were negative.

Commonly misidentified lines
(See ICLAC register)

N/A

Animals and other research organisms

Policy information about studies involving animals; ARRIVE guidelines recommended for reporting animal research, and Sex and Gender in Research

Laboratory animals

Ncr1-iCreERT2 mice were developed in the Fehniger lab on the C57BL/6J background. Mice were bred to Rosa26 YFPLSL knock-in mice (The Jackson Laboratory, stock no. 006148) as well as Eomes floxed mice (The Jackson Laboratory, stock no. 017293) and Tbet floxed mice (The Jackson Laboratory, #022741 (RRID:IMSR_JAX:022741).

Wild animals	N/A
Reporting on sex	N/A
Field-collected samples	N/A
Ethics oversight	Mice were bred and maintained in specific pathogen-free housing, and experiments were conducted in accordance with the guidelines of and with the approval of the Washington University Washington University Institutional Animal Care and Use Committee and with the approval of the IACUC (20170129). Mice under survival study were euthanized if mice lost more than 20% of their body weight. Mice that display paralysis were euthanized. In all cases, any moribund animal were euthanized.

Note that full information on the approval of the study protocol must also be provided in the manuscript.

Plants

Seed stocks	N/A
Novel plant genotypes	N/A
Authentication	N/A

Flow Cytometry

Plots

Confirm that:

- [x] The axis labels state the marker and fluorochrome used (e.g. CD4-FITC).
- [x] The axis scales are clearly visible. Include numbers along axes only for bottom left plot of group (a 'group' is an analysis of identical markers).
- [x] All plots are contour plots with outliers or pseudocolor plots.
- [x] A numerical value for number of cells or percentage (with statistics) is provided.

Methodology

Sample preparation	Sample preparationCardiac puncture was performed to collect blood. Spleens and inguinal lymph nodes were crushed with the back of syringe plunger through a 70um filter to generate a single cell suspension. Lungs were first digested with collagenase and DNAse prior to further processing. BM was harvested by flushing one femur from each mouse with a 23-gauge needle. Whole livers were homogenized with glass douncers followed by percoll gradient to isolate lymphocytes. All tissues were ACK lysed to remove red blood cells. Zombie dye was incubated for 15 minutes at room temperature and surface staining was performed at 4 degrees for 20 min-1 hour. Intracellular staining was performed in permeabilization buffer following fixation/permeabilization with kits from BD Biosciences (for IFN-γ staining) or Invitrogen eBioscience Fix/Perm kit. When using the eBioscience Fix/Perm kit which quenches YFP: after surface staining cells were briefly fixed in 1% PFA for 2 minutes at room temperature prior to fixation/permeabilization in order to preserve YFP when intracellular proteins were assessed. For T-box TF staining, cells were incubated in eBioscience 1X Fix/Perm overnight at 4 degrees in the dark, then the next day proceeded to staining overnight in 1X Perm Buffer at 4 degrees in the dark.
Instrument	Flow cytometry data were collected on the Beckman Coulter Gallios, Thermo Fisher Attune Nxt, or Cytek Northern Light flow cytometers. Cell sorting was performed on a BD FACS Aria II or Cytek Aurora sorter
Software	Data was analyzed using FlowJo (Treestar) software.
Cell population abundance	Cell sorting was performed on a BD FACS Aria II or Cytek Aurora sorter to greater than 95% purity. Post-sort purity check was done by sampling from the sorted samples.
Gating strategy	Samples were first gated for singlets then a lymphocyte gate was drawn in 2D plot of FSC-A vs SSC-A. Then CD45+ cells were gated before assessing NK cells via NK1.1 and NKp46 expression.

- [x] Tick this box to confirm that a figure exemplifying the gating strategy is provided in the Supplementary Information.
