# Supplementary material for: Inducible T-bet deletion reveals tissue-specific requirements in NKp46+ ILC immunobiology and response to murine cytomegalovirus
Source: Nat Commun. 2026 Jun 18;17:7708. doi: 10.1038/s41467-026-74478-y (PMC13434699; doi:10.1038/s41467-026-74478-y)
Supplement: Supplementary file 3 [file 41467_2026_74478_MOESM3_ESM.pdf]

nature portfolio

Peer Review File

Inducible T-bet deletion reveals tissue-specific requirements in NKp46+ ILC immunobiology and response to murine cytomegalovirus

Corresponding Author: Dr Todd Fehniger

This file contains all reviewer reports in order by version, followed by all author rebuttals in order by version.

Version 0:

Reviewer comments:

Reviewer #1

(Remarks to the Author)
The authors report the generation of a tamoxifen-inducible model of T-bet deficiency, specifically in NCR1+ cells. Using this model, they revisit the role of T-bet in the survival, phenotype and function of NCR1+ cells in different organs. The main strength of the article is that the role of T-bet in the maintenance of mature NK cells had never been studied using such a refined model. Its primary weakness is that this role does not significantly differ from its role in NK cell development. Another interesting aspect of the paper is that its data align with the anticipated role of T-bet and the majority of findings in the field, while contradicting a previous study that employed a different inducible T-bet deletion model and unexpectedly reported minimal impact of T-bet on the homeostasis of mature NK cells1. However, several points deserve attention from the authors:

1 – The title should be changed to highlight the use of the conditional-inducible model of T-bet deletion

2 – While I agree that the model used by the authors is refined compared to previous ones, the authors should refrain from using sentences like "little is known about the role of these T-box TFs to orchestrate the biology of mature NK cells" or "the redundancy (or lack of thereof) between T-bet and Eomes to sustain mature NK cell programs is unknown". In fact, as the authors know very well, both TFs are probably the best-known TFs in NK cell biology, in particular from CHIP, ATAC and other epigenetic studies in T-bet/Eomes sufficient mice, and their respective roles starts to be well understood. The gap of knowledge addressed by the authors should therefore be more accurately defined.

3 – The sentence at the end of page 2 of the results "despite a mostly intact NK cell compartment…) is wrong: the authors show multiples NK cell alterations in these mice, which likely explain the poor control of B16 metastases.

4 – In figures 3 and 4, the authors addressed the role of T-bet and Eomes in NK/ILC1s from different tissues. They conclude that T-bet is not essential for the maintenance of liver NK cells and that of SI ILC1s. However, a more detailed phenotypic analysis should be performed to confirm this point. In particular, stage IV NK cells are likely affected by T-bet deficiency. Moreover, the NK/ILC1 phenotype was not studied beyond 7 days after Tamoxifen injection. Longer time points should be used since a previous study found that the role of T-bet in these subsets was only evident after 3 weeks of tamoxifen usage in a different model2. This reference should also be cited and discussed.

5 – In the discussion, the authors consider the absence of T-bet role in NK cell homeostasis in the BM. I suggest the authors discuss the very low expression of T-bet in this compartment, as previously reported in our study3.

6 – In the discussion (bottom of third page), the authors contradict the conclusions from Weizman's study on the role of ILC1s in the defense against MCMV. However, the authors should be cautious and indicate that ILC1s are not required "in the experimental conditions used in the present manuscript".

References

1. Madera, S. et al. Cutting Edge: Divergent Requirement of T-Box Transcription Factors in Effector and Memory NK Cells. J. Immunol. Baltim. Md 1950 200, 1977–1981 (2018).
2. Schroeder, J.-H. et al. Sustained Post-Developmental T-Bet Expression Is Critical for the Maintenance of Type One Innate Lymphoid Cells In Vivo. Front. Immunol. 12, (2021).
3. Daussy, C. et al. T-bet and Eomes instruct the development of two distinct natural killer cell lineages in the liver and in the bone marrow. J. Exp. Med. 211, 563–577 (2014).

Reviewer #2

(Remarks to the Author)
In this manuscript, the authors use in vivo inducible deletion of T-bet to study the role of this transcription factor in regulating the activity of mature NKp46+ innate lymphoid cells. The group reports that T-bet deletion in mature NK cells results in apoptosis at steady-state and a reduction in cell numbers in various tissues during homeostasis. Functional assays reveal that T-bet deficiency impairs cytokine production in Stage II NK cells and compromises immunity against various tumor models. Expanding their analysis to other NKp46+ cell types, the authors show that T-bet is also crucial for maintaining liver-resident ILC1s and NKp46+ ILC3s in the small intestinal lamina propria. Additionally, using murine cytomegalovirus (MCMV) as a model for infection, the authors uncover a distinct role for Eomes in mature NK cells, with its deficiency, but not that of T-bet, diminishing antiviral immunity and reducing Group 1 ILCs across tissues. While the manuscript is well-written and most experiments are executed effectively, several major concerns remain. Notably, the broader implications of these findings are not thoroughly explored. Below, the major points of critique are outlined.:
Main points:
• In Extended Data Figure 1c, a small but significant population of NK cells did not acquire YFP, indicating that T-bet was not deleted in these cells. The authors are strongly encouraged to reanalyze the data by gating on YFP-negative and YFP-positive NK cells separately in most figures. Comparing these two subsets within the same animals would provide stronger evidence for the reported findings.

• In Fig. 1, while the authors say it's unlikely that the induced T-bet deletion influenced NK cell maturation, it remains a possibility. Thus, the certainty with which the authors conclude that the drop in mature NK cells is a result only of apoptosis in this sub-population does not seem completely justified. Could the authors perform cell sorting of Stage II, III, and IV splenic NK cells, induce T-bet deletion in vitro, and evaluate viability, apoptosis, changes in maturation status? Studying each population in isolation in this way would allow the group to circumvent the potentially confounding variables in their in vivo study. Should the conclusion still hold, the authors should minimally propose an hypothesis on why T-bet deficiency within mature NK cells resulted in the loss of Stage IV NK in some tissues spleen, blood, lung but not in BM and LN. In addition, it is unclear whether CD49a+DX5- cells were excluded from the analysis. This is an important consideration, as the plasticity among different ILC1 subsets could influence the data and interpretations. Clarification or reanalysis is recommended to account for this potential confounding factor.

• In Figure 2, the authors report that the bulk population of Ncr1-Tbx21Δ/Δ NK cells produces similar levels of IFN-γ as control NK cells. However, Stage II NK cells, but not Stages III and IV, show significantly reduced IFN-γ production in response to IL-12 and IL-15. This raises the question of whether another stage might have increased IFN-γ production to compensate, resulting in unchanged overall levels. Further clarification or analysis is needed to address this discrepancy. Additionally, IL-12/IL-18 is typically used in such assays, so the use of IL-12/IL-15 in this context is unusual and could potentially explain discrepancies with previous reports. The authors should clarify this choice and discuss how it may influence their findings. In Figure 2c, the authors report that NK cell degranulation in response to YAC-1 cells is significantly reduced in Ncr1-Tbx21Δ/Δ NK cells. However, the analysis does not differentiate between the various NK cell stages. Including stage-specific analysis would provide a more detailed understanding of how T-bet deletion affects NK cell function.

• Figure 6 is difficult to interpret due to inconsistent axis labeling and data presentation. For example, the authors present data as “percent of lymphocytes” (6d-e) and “frequency of lymphocytes” (6g-k), rather than as “percent of NK cells” as done previously, making comparisons challenging. Furthermore, the data in Figure 6e suggests that ~0.1% of lymphocytes are KLRG1+/CD27+ NK cells after infection, which seems unusually low. In Figure 6h, if the values are decimals rather than percentages, it is unclear how a population can exceed 1.0. The authors should clarify these points and consider presenting the actual number of NK cells, which would be more informative. Additionally, in Figure 6b, T-bet KO mice appear to be somewhat protected from MCMV infection relative to WT mice, even if the difference is not statistically significant. The authors could speculate on potential mechanisms for this observation. For example, is there a specific NK/ILC1 population that is lost in T-bet KO mice that might contribute to immune pathology during infection? Addressing this could enhance the interpretation of the data.

Minor points:
• Because the lung contains many vasculature-associated cells, is the reduction observed in total lung NK (and in Stage IV NK cells from lung) merely be a function of a reduction in blood NK cells? Perhaps IV labeling would help to determine whether tissue-resident cells are reduced as well.

• In the discussion, could the authors expand speculation as to why Stage IV NK cells in the bone marrow are found at similar/higher levels in T-bet/Eomes dKO mice despite the opposite being found in all other tissues? Might Eomes play a role in promoting egress from the BM in the most mature NK cells?

• Is there a reason why the authors use tamoxifen chow in Fig. 6 as opposed to i.p. injections as performed in all previous experiments?

• Others: Figure 1g should be percent on the axis – Figure 5 frequency of lymphocytes

Reviewer #3

(Remarks to the Author)
In their manuscript, “Tissue-specific requirement for T-bet in NKp46+ innate lymphoid cell homeostasis, function, and response to MCMV,” Wong et al. use tamoxifen-inducible NKp46-Cre-ERT2 mice [PMID: 34521844] to investigate the roles of the T-box transcription factors T-bet and Eomes in mature NKp46+ innate lymphoid cells (NK cells, ILC1s, and NKp46+ ILC3s). Through conditional and inducible deletion of Tbx21 in fully developed NKp46+ cells, the authors uncover tissue- and subset-specific dependencies on the continuous expression of this transcription factor. Their findings demonstrate that sustained T-bet expression is critical for the survival of liver-resident ILC1s but dispensable for ILC1s in the small intestine, highlighting a tissue-specific role for T-bet in ILC1 homeostasis. In contrast, while T-bet deletion did not affect NK cells or ILC1s in the small intestine, it markedly compromised the survival of NKp46+ ILC3s, demonstrating subset-specific roles of T-bet in shaping NKp46+ ILC homeostasis. Furthermore, inducible deletion of T-bet modestly affected the survival of mature (stage IV) NK cells in the spleen and lung, leading to reduced anti-tumor immune responses in the lungs of mice injected with B16-F10 melanoma. Lastly, by contrasting the effects of Tbx21 and Eomes deletion in NKp46-expressing cells, the authors demonstrate that NK cells, rather than ILC1s, are essential for mounting a protective response against MCMV infection.

Major Concerns:

While the study highlights some new results, its overall impact is undermined by a lack of conceptual novelty, insufficient exploration of broader implications, and an overly descriptive presentation.

The use of an inducible conditional knockout model allows for precise dissection of T-bet and Eomes functions in fully developed NK cells and ILCs, overcoming limitations of previous germline knockout or constitutive NKp46-Cre+ conditional knockout mice. Additionally, the authors provide a phenotypic analysis of the transcriptional and functional consequences of T-bet loss in NKp46+ ILCs across multiple tissues. These aspects provide a strong foundation for mechanistically dissecting tissue-specific dependencies of T-box transcription factors in innate lymphoid cells.

Despite these strengths, the manuscript struggles to advance the field conceptually. Many of the findings reiterate known roles of T-bet and Eomes in NK cells, such as their contributions to NK cell maturation and cytotoxicity [PMID: 34521844, PMID: 29440505, PMID: 24516120]. Although the tissue-specific differences observed in this study are interesting, they represent incremental advances rather than groundbreaking insights. For instance, the observation that liver ILC1s require sustained T-bet expression while small intestine ILC1s do not is intriguing but insufficiently explored within the broader framework of immune regulation.

The discussion section is another area where the manuscript falls short. It is wordy, unfocused, and often reiterates results rather than synthesizing them into a coherent narrative.

Overall, while the technical execution is commendable, the manuscript falls short of conceptual depth. The study would benefit from providing mechanistic insights and presenting a more cohesive narrative to highlight its contribution to the field.

Version 1:

Reviewer comments:

Reviewer #1

(Remarks to the Author)
I thank the authors for addressing my comments, the paper is now suitable for publication.

Reviewer #2

(Remarks to the Author)
In this revised version, the authors have seriously addressed the major issues raised in the initial review, and the manuscript has significantly improved. I have no further concerns.

Reviewer #3

(Remarks to the Author)
The revised manuscript is improved. The additional experiments address several of the specific issues raised in the initial review, and the study is now better supported than in its original form. The added analysis of liver NK-cell maturation, the longer-term assessment of SI LP populations, and the expanded functional studies make the dataset more complete.

My main concern, however, remains one of conceptual significance rather than technical execution.
The major strength of the study is the inducible, NKp46-restricted deletion strategy, which allows the authors to interrogate the requirement for sustained T-bet expression after development rather than infer function from constitutive or developmental loss-of-function models. That is an important technical refinement and a useful experimental system. But a better system does not, by itself, produce a proportionately greater conceptual advance.

The manuscript still reads largely as a catalog of tissue- and subset-specific phenotypes following inducible deletion of T-bet or Eomes. The authors show effects in spleen, blood, lung, liver, SI LP, and in the context of MCMV infection. These findings are of interest. But the presentation continues to treat the breadth of the experimental survey as a substitute for a unifying biological insight. It is not. A longer list of affected compartments, phenotypes, or assays does not in itself establish novelty.

To my mind, the strongest aspect of the revised manuscript is the MCMV analysis. Here the authors can reasonably argue that, using a temporally controlled genetic model, they revisit a prior literature emphasizing ILC1-mediated protection and instead conclude that Eomes-dependent NK cells, rather than T-bet-dependent ILC1s, are the dominant protective compartment under their experimental conditions. That is a meaningful point. However, even here the claim should remain carefully bounded to the model and conditions used, as the authors now acknowledge.

Outside of that aspect, the biological conclusions remain relatively incremental. The paper supports the view that sustained T-bet expression contributes to the maintenance of selected mature NK/ILC compartments in a tissue-specific manner, while Eomes has a more prominent role in antiviral protection in the MCMV setting examined here. Useful as these observations are, the manuscript still does not provide a mechanistic framework that explains the tissue specificity it documents. Why stage IV NK cells are selectively affected in some compartments and not others, why bone marrow NK cells are spared, and why liver and intestinal ILC compartments differ in kinetics and extent of dependence all remain unresolved.

Accordingly, my concern is not that the manuscript lacks data. It is that the conceptual advance remains narrower than the authors claim. The work is stronger technically than intellectually. The revisions improve the rigor of the manuscript, but they do not fully change its basic character from a careful phenotypic dissection in a refined model to a study delivering a broad mechanistic or conceptual advance.

Open Access This Peer Review File is licensed under a Creative Commons Attribution 4.0 International License, which permits use, sharing, adaptation, distribution and reproduction in any medium or format, as long as you give appropriate credit to the original author(s) and the source, provide a link to the Creative Commons license, and indicate if changes were made.
In cases where reviewers are anonymous, credit should be given to 'Anonymous Referee' and the source.
The images or other third party material in this Peer Review File are included in the article’s Creative Commons license,

unless indicated otherwise in a credit line to the material. If material is not included in the article's Creative Commons license and your intended use is not permitted by statutory regulation or exceeds the permitted use, you will need to obtain permission directly from the copyright holder.
To view a copy of this license, visit https://creativecommons.org/licenses/by/4.0/

Point-by-Point Response to Reviewer Comments

We thank the editor and reviewers for their careful reading of this manuscript, and insightful comments and questions. We have carefully addressed each comment below and include experimental data to support our response whenever possible. This revision process did take some time, since the experiments required breeding complex genotypes. After revising the manuscript based on your critique, we believe the manuscript has substantially improved, and we hope it is acceptable for publication.

REVIEWER COMMENTS

Reviewer #1 (Remarks to the Author):

The authors report the generation of a tamoxifen-inducible model of T-bet deficiency, specifically in NCR1+ cells. Using this model, they revisit the role of T-bet in the survival, phenotype and function of NCR1+ cells in different organs. The main strength of the article is that the role of T-bet in the maintenance of mature NK cells had never been studied using such a refined model. Its primary weakness is that this role does not significantly differ from its role in NK cell development. Another interesting aspect of the paper is that its data align with the anticipated role of T-bet and the majority of findings in the field, while contradicting a previous study that employed a different inducible T-bet deletion model and unexpectedly reported minimal impact of T-bet on the homeostasis of mature NK cells1. However, several points deserve attention from the authors:

We appreciate your comments and careful read and agree that providing clear data in the very best model systems to support the role of T-bet (and Eomes) in mature NK cell homeostasis is an important endeavor in the field. Thank you for pointing out the strength of our model and the importance of our new findings related to the *in vivo* role of T-bet for mature NK cell and other NCR1$^+$ ILC homeostasis.

1 – The title should be changed to highlight the use of the conditional-inducible model of T-bet deletion

Thank you for this suggestion. We have changed the title accordingly.

Original: Tissue-specific requirement for T-bet in NKp46+ innate lymphoid cell homeostasis, function, and response to MCMV

Revised: Inducible deletion of T-bet reveals tissue-specific requirements in NKp46+ ILC immunobiology and response to MCMV

2 – While I agree that the model used by the authors is refined compared to previous ones, the authors should refrain from using sentences like “little is known about the role of these T-box TFs to orchestrate the biology of mature NK cells” or “the redundancy (or lack of thereof) between T-bet and Eomes to sustain mature NK cell programs is unknown”. In fact, as the authors know very well, both TFs are probably the best-known TFs in NK cell biology, in particular from CHIP, ATAC and other epigenetic studies in T-bet/Eomes sufficient mice, and

their respective roles starts to be well understood. The gap of knowledge addressed by the authors should therefore be more accurately defined.

Thank you for this comment, we certainly acknowledge that T-bet and Eomes have been studied using CHIP-seq, ATAC-seq and other epigenetic approaches, as well as the well-defined impact of these TFs during NK cell development. Our intention was to plainly point out the gap in our knowledge related to ILC-specific, intrinsic inducible loss-of-function after normal development. In our view, this is a critical aspect of mature NK cell biology that requires the complex models developed and employed in the current study. We view this approach to be complementary to the many other studies of T-bet and Eomes in the field and have updated the writing to be more precise about the gap addressed by this study. This included updating the first sentence of the Abstract, and the Introduction. We also concisely reference a large number of studies referred to by the reviewer in the Introduction, acknowledging what is known: "Studies of T-bet and Eomes have shown overlapping targets at the genomic level due to their highly homologous T-box DNA binding domain, but they have also been demonstrated to have unique functions.[6,15–17]" Finally, we've rewritten parts of the **Discussion** to better synthesize our findings with the literature available in the field.

For the Abstract:

Original: The role of the T-box transcription factors T-bet and Eomes in innate lymphoid cells (ILCs) beyond their development is not well understood.

Revised: The requirement for the T-box transcription factors T-bet and Eomes in innate lymphoid cells (ILCs) beyond their development is not well understood.

For the Introduction:

Original: Hence, little is known about the role of these T-box TFs to orchestrate the biology of mature NK cells.

Revised: "Hence, the requirement of these T-box TFs for mature NK cell homeostasis and function (after development) remains unclear."

Original: These findings caution against using broad T-box TF loss-of-function mouse models which have impaired NK development, non-NK/ILC effects, and/or ex vivo or adoptive transfer models.

Revised: These findings caution against using broad T-box TF loss-of-function mouse models which have impaired NK development, non-NK/ILC effects, and/or ex vivo or adoptive transfer models, and highlight the importance of using conditional, inducible models to study the intrinsic role of T-box TFs in mature NK cells.

3 – The sentence at the end of page 2 of the results "despite a mostly intact NK cell compartment…) is wrong: the authors show multiples NK cell alterations in these mice, which likely explain the poor control of B16 metastases.

Thank you for your comment, our intent here was to point out that stage II-III NK cells were intact, as were many aspects of the NK functional response, highlighting that phenotypes could be observed with only partial impact on mature NK cells. For clarity, we have revised this sentence:

"Thus, selective loss of Stage IV NK cells in the blood and lung, along with decreased degranulation and killing, were sufficient to compromise control of B16-F10 metastases."

4 – In figures 3 and 4, the authors addressed the role of T-bet and Eomes in NK/ILC1s from different tissues. They conclude that T-bet is not essential for the maintenance of liver NK cells and that of SI ILC1s. However, a more detailed phenotypic analysis should be performed to confirm this point. In particular, stage IV NK cells are likely affected by T-bet deficiency.

Thank you for your suggestion, which is well reasoned based on the observation that Stage IV NK cells were affected in other tissues. In new experiments, we have performed stage-specific analysis of liver NK cells

(Extended Figure 3, a-b). Although there was a significant decrease in Stage IV NK cells (as postulated by the Reviewer), the difference in overall total NK cell numbers was not significantly altered, since Stage IV NK cells represent only a fraction of overall NK cells. To further expand on this comparison, we also performed functional analyses and measured IFN-γ production and degranulation of liver NK cells (Extended Figure 3, c-f), which was similar to that of splenic NK cells. The narrative of the Results has been revised to reflect these new data.

Moreover, the NK/ILC1 phenotype was not studied beyond 7 days after Tamoxifen injection. Longer time points should be used since a previous study found that the role of T-bet in these subsets was only evident after 3 weeks of tamoxifen usage in a different model2. This reference should also be cited and discussed.

Thank you for your comment and the opportunity to clarify our objectives and experimental design. In our study, we performed the analysis after one week to elucidate the immediate, intrinsic effect of loss of T-bet on NKp46+ NK/ILC subsets. We were concerned that at later timepoints ongoing NK cell maturation and replenishment arising from non-Cre exposed immature NK cells would confound our evaluation. Based on this Day 6 (post-tamoxifen) analysis, our findings showed that in the liver, ILC1s were significantly decreased, whereas only Stage IV NK cells were decreased from controls. In addition, the SI LP showed no change in NK cells or ILC1s, but did show a significant decrease in NKp46+ ILC3s.

To address this comment, we performed additional experiments waiting until Day 22 for analysis of the longer-term impact of T-bet deletion on mature ILC subsets (**Extended Data Figure 5**). Due to the confounding factor of ongoing NK cell turnover over the time course of weeks, we found that Cre-exposed (YFP+) NK cells were a minority of NK cells at Day 22 even in control mice, and were therefore not evaluated (**Extended Figure 5b**). In the liver, we observed a prolonged decrease in ILC1s. In the SI LP, at day 22 we observed a reduction in ILC1s, though not as drastic as the complete loss of ILC1s in the liver, as well as an ongoing decrease in NKp46+ ILC3s.

Thus, the only major difference for Day 22 post-tamoxifen was a new decrease in SI LP ILC1s. We have added this finding to our **Results** narrative. Considering that liver ILC1s were reduced in the liver within 7 days, and NKp46+ ILC3s were similarly reduced in the SI LP within 7 days, the much longer time requirement to observe a reduction in ILC1s in the SI LP may be due to a different mechanism or partial rescue by an alternative transcriptional program. We've updated our conclusions to reflect that short-term (1 week) SI LP ILC1s were preserved, whereas over the long-term (3 weeks) they did exhibit a reduced number.

We have also expanded the Discussion involving Schroeder et al., 2021 (**ref 33 in revised manuscript**).

5 – In the discussion, the authors consider the absence of T-bet role in NK cell homeostasis in the BM. I suggest the authors discuss the very low expression of T-bet in this compartment, as previously reported in our study3.

Thank you for this comment, we have expanded the **Discussion** (paragraph 2) and specifically cited the reduced T-bet expression in the bone marrow (reference 10 in the revised manuscript), which is consistent with our finding that T-bet deletion did not impact the bone marrow NK cell compartment.

Discussion (Paragraph 3) "Notably, T-box TF expression levels have been shown to be lower in NK cells in the BM, compared to those in the periphery, which also suggests the potential of other TFs supporting BM NK cells.[10]"

6 – In the discussion (bottom of third page), the authors contradict the conclusions from Weizman's study on the role of ILC1s in the defense against MCMV. However, the authors should be cautious and indicate that ILC1s are not required "in the experimental conditions used in the present manuscript".

Thank you for your comment. We have included the caveat of different experimental models in our discussion to clarify we are utilizing a distinct model system.

Discussion (Paragraph 6): "Thus, our data indicates that despite ILC1's rapidly producing IFN-γ during early MCMV infection (as demonstrated by Weizman et al.), they are not required for normal MCMV host defense in the conditions used in our experiments.[28]"

Reviewer #2 (Remarks to the Author):

In this manuscript, the authors use in vivo inducible deletion of T-bet to study the role of this transcription factor in regulating the activity of mature NKp46+ innate lymphoid cells. The group reports that T-bet deletion in mature NK cells results in apoptosis at steady-state and a reduction in cell numbers in various tissues during homeostasis. Functional assays reveal that T-bet deficiency impairs cytokine production in Stage II NK cells and compromises immunity against various tumor models. Expanding their analysis to other NKp46+ cell types, the authors show that T-bet is also crucial for maintaining liver-resident ILC1s and NKp46+ ILC3s in the small intestinal lamina propria. Additionally, using murine cytomegalovirus (MCMV) as a model for infection, the authors uncover a distinct role for Eomes in mature NK cells, with its deficiency, but not that of T-bet, diminishing antiviral immunity and reducing Group 1 ILCs across tissues. While the manuscript is well-written and most experiments are executed effectively, several major concerns remain. Notably, the broader implications of these findings are not thoroughly explored. Below, the major points of critique are outlined.:

Main points:

• In Extended Data Figure 1c, a small but significant population of NK cells did not acquire YFP, indicating that T-bet was not deleted in these cells. The authors are strongly encouraged to reanalyze the data by gating on YFP-negative and YFP-positive NK cells separately in most figures. Comparing these two subsets within the same animals would provide stronger evidence for the reported findings.

Thank you for the opportunity to explain our choice of control mice. In most situations for the Ncr1-iCreERT2 x Rosa^{YFP} reporter (x T-bet fl/fl or Eomes fl/fl), the percentage of YFP- NK cells and ILCs is very small, resulting in low numbers of total events, leading to unreliable gating and cell frequencies, especially when evaluating for NK cell stages. In addition, the small percentage of YFP- NK cells do not experience Cre-mediated DNA breaks, resulting in a potential confounder. Finally, as we follow these YFP- cells over time, they include all newly maturing NK cells, leading to biases in analysis. For these reasons, we instead use Ncr1-iCreERT2 x Rosa26^{YFP} reporter mice as controls, treated with the same tamoxifen, bred in the same colony, and used as age- and sex-matched controls. This is the same approach we used in our previous analysis of Eomesodermin floxed mice and Acly floxed mice with the conditional, inducible iCre (e.g., PMID: 32492428, PMID: 40853248).

• In Fig. 1, while the authors say it's unlikely that the induced T-bet deletion influenced NK cell maturation, it remains a possibility. Thus, the certainty with which the authors conclude that the drop in mature NK cells is a result only of apoptosis in this sub-population does not seem completely justified. Could the authors perform cell sorting of Stage II, III, and IV splenic NK cells, induce T-bet deletion in vitro, and evaluate viability, apoptosis, changes in maturation status? Studying each population in isolation in this way would allow the group to circumvent the potentially confounding variables in their in vivo study.

We appreciate this comment and technical challenges of separating apoptosis vs. cell loss from maturation defects in vivo in this experimental setting.

We performed the experiment requested by the reviewer by sorting stage II/III NK cells from T-bet WT and T-bet$^{\Delta/\Delta}$ mice, treated with tamoxifen in vitro to induce deletion of the alleles, and maintained the NK cells with IL-15 to assess for maturation. However, even in the WT setting, this in vitro culture approach failed to result in the maturation of stage IV NK cells from stage II/III NK cells over the course of 9 days, which is the maximal time for murine NK cells to survive in vitro in our experience. This technical issue therefore precludes addressing NK cell maturation changes in vitro (Reviewer Figure 1).

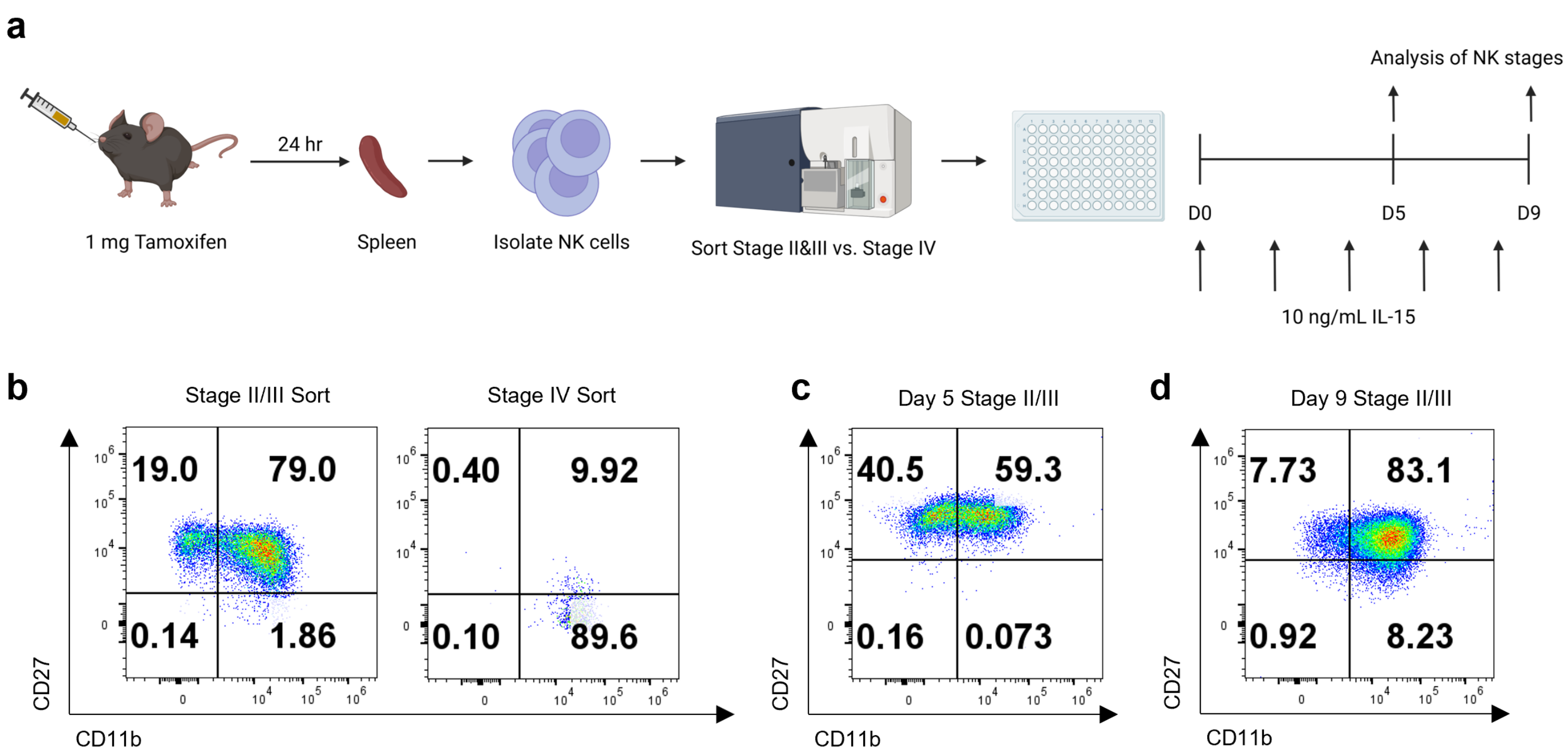

Reviewer Figure 1. Sorted Stage II/III splenic NK cells do not mature to Stage IV *in vitro*. a, Schema of in vitro experiment. Ncr1-iCreERT2 control mice were treated with 1 mg tamoxifen by oral gavage, then 24 hours later, splenic NK cells were isolated and stage II/III NK cells were sorted and plated with 10 ng/mL IL-15 to induce in vitro differentiation. **b**, post-sort analysis of Stage II/III vs. IV NK cells ($CD3^{-}NK1.1^{+}NKp46^{+}$). **c-d**, NK stage analysis c, 5-days post sort and d, 9-days post sort.

Studies that established the NK cell Stage II->III->IV differentiation (PMID: 19234143) sorted each stage of NK cells, transferred each stage into an NK cell-deficient mouse, then analyzed the resulting stage in vivo output on day 14. We considered performing the same experiment, but realized that we could not attribute the expected Stage IV NK cell decreases in this model to maturation vs. survival. Since our original data shows that Stage IV NK cells are lost quickly (after 2-4 days) and have increased Caspase 3/7 activity (suggestive of apoptosis), we concluded that apoptosis is a contributing mechanism. Since we cannot experimentally evaluate in vitro murine NK cells stage differentiation, we've modified our conclusions and the **Discussion** (Paragraph 2) to reflect this possibility, as suggested by the Reviewer.

Should the conclusion still hold, the authors should minimally propose an hypothesis on why T-bet deficiency within mature NK cells resulted in the loss of Stage IV NK in some tissues spleen, blood, lung but not in BM and LN.

Thank you for your comment, we have expanded our discussion of tissue-specific impact of T-bet deficiency on Stage IV NK cell homeostasis in the **Discussion** (Paragraph 3).

In addition, it is unclear whether CD49a+DX5- cells were excluded from the analysis. This is an important consideration, as the plasticity among different ILC1 subsets could influence the data and interpretations. Clarification or reanalysis is recommended to account for this potential confounding factor.

Thank you for your comment. CD49a+DX5- cells were only excluded in the analysis in the liver, as their numbers are very low in other tissues. We have updated the figure legends to explicitly reflect gating strategies to indicate when the CD49a+DX5- gate was used. We have also re-analyzed the data for splenic NK cells but did not observe any changes in the results (Reviewer Figure 2). Based on these findings and re-analysis, we do not expect this to be a confounder.

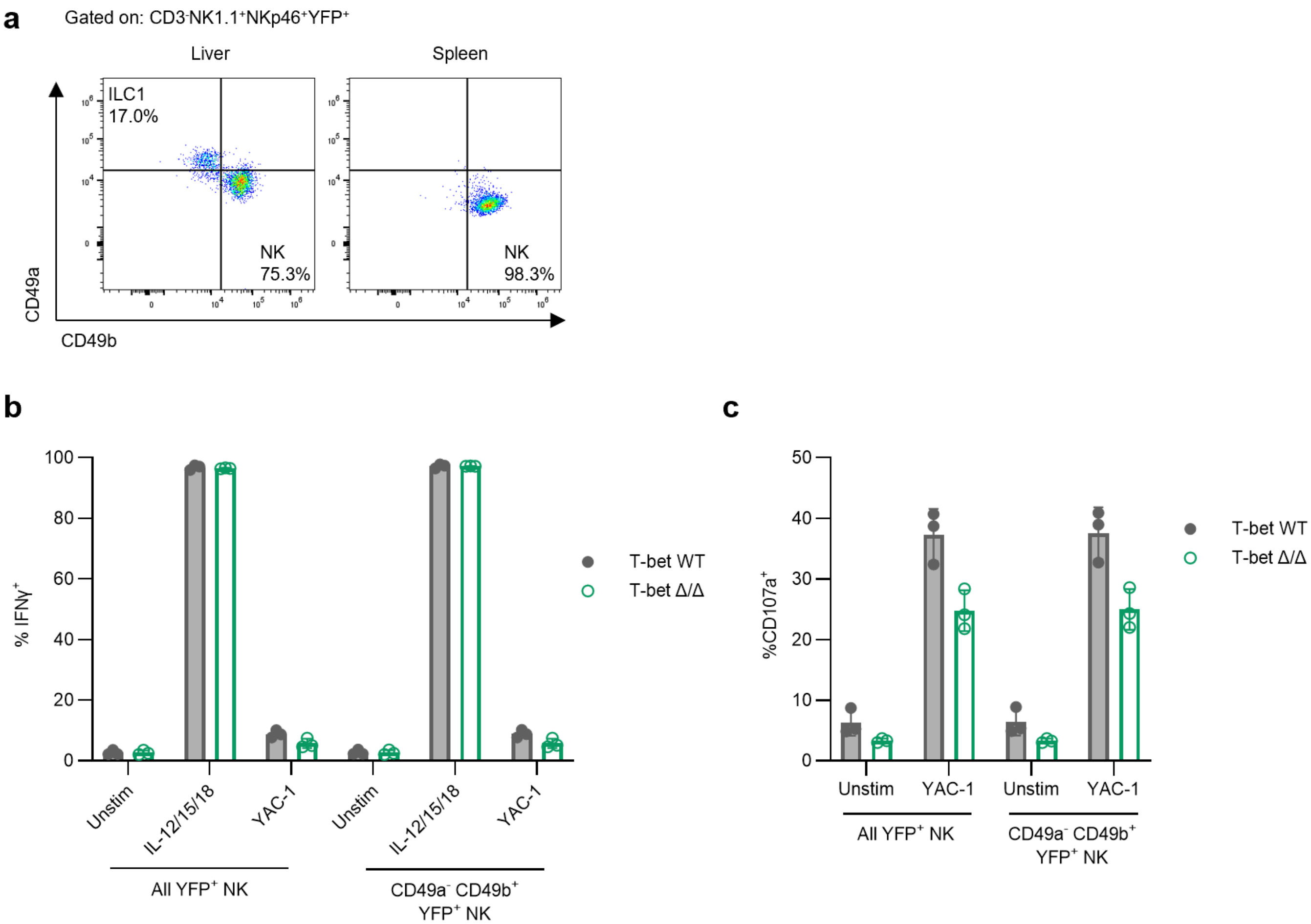

Reviewer Figure 2. Most splenic NK1.1⁺NKp46⁺ cells are CD49b⁺ CD49a⁻. **a**, Representative flow plot of CD3⁻ NK1.1⁺NKp46⁺ from liver vs. spleen. **b-c** side-by-side comparison of b, IFN-γ production and c, CD107a⁺ population within splenic NK calculated using the manuscript's gating strategy (left) vs. additional gating on CD49b⁺ CD49a⁻ population (right). Adding this gate did not alter the results or conclusions.

• In Figure 2, the authors report that the bulk population of Ncr1-Tbx21Δ/Δ NK cells produces similar levels of IFN-γ as control NK cells. However, Stage II NK cells, but not Stages III and IV, show significantly reduced IFN-γ production in response to IL-12 and IL-15. This raises the question of whether another stage might have increased IFN-γ production to compensate, resulting in unchanged overall levels. Further clarification or analysis is needed to address this discrepancy. Additionally, IL-12/IL-18 is typically used in such assays, so the use of IL-12/IL-15 in this context is unusual and could potentially explain discrepancies with previous reports. The authors should clarify this choice and discuss how it may influence their findings.

Thank you for this comment and the opportunity to clarify our results. The difference in IFN-γ between T-bet WT and T-bet$^{\Delta\Delta}$ for Stage II NK cells is evident only in response to cytokines (IL-12 plus IL-15). For Stages III and IV, which comprise the majority of NK cells, there was no difference observed. To address the question of overall IFN-γ, we show in Figure 2a of the revised manuscript that when gating on all YFP+ NK cells, the difference in Stage II NK does not result in differences in the overall NK cell production of IFN-γ in response to IL-12 plus IL-15.

In addition, thank you for the opportunity to clarify our choice of cytokines to stimulate NK cell IFN-γ production. Our group uses both IL-12 plus IL-15 and IL-12 plus IL-15 plus IL-18 to induce IFN-γ in NK cells, based on prior reports that these induce different degrees of IFN-γ response (e.g. PMID 10201989). We utilized IL-12 plus IL-15 to observe an intermediate IFN-γ response, (e.g., 60% positive NK cells in Figure 2a), allowing for detection of increases or decreases between control and experimental conditions. In contrast, we utilize IL-12 plus IL-15

plus IL-18 when evaluating for a maximal IFN-γ response. To address the comment, we have performed additional experiments using IL-12 plus IL-15 plus IL-18 stimulation and observed increased IFN-γ production overall, with similar findings regarding the reduced stage II IFN-γ production (**Extended Figure 1, g-h**) with overall increased IFN-γ production with these more potent stimuli.

In Figure 2c, the authors report that NK cell degranulation in response to YAC-1 cells is significantly reduced in Ncr1-Tbx21Δ/Δ NK cells. However, the analysis does not differentiate between the various NK cell stages. Including stage-specific analysis would provide a more detailed understanding of how T-bet deletion affects NK cell function.

Thank you for your comment. To address this, we have included the stage specific analysis of NK cell degranulation in response to YAC-1 target cells in **Figure 2d**, which reveals that T-bet deficiency reduced Stage III and Stage IV (but not Stage II) degranulation. This has been added to the **Results** narrative.

• Figure 6 is difficult to interpret due to inconsistent axis labeling and data presentation. For example, the authors present data as “percent of lymphocytes” (6d-e) and “frequency of lymphocytes” (6g-k), rather than as “percent of NK cells” as done previously, making comparisons challenging. Furthermore, the data in Figure 6e suggests that ~0.1% of lymphocytes are KLRG1+/CD27+ NK cells after infection, which seems unusually low. In Figure 6h, if the values are decimals rather than percentages, it is unclear how a population can exceed 1.0. The authors should clarify these points and consider presenting the actual number of NK cells, which would be more informative.

Thank you for your comment and the opportunity to clarify the data presentation. We now present all of the data as percentage of lymphocytes to allow the reader to understand the differences in overall frequency between the various liver NK and ILC populations as indicated in the figure top labels. For Figure 6g, control and T-bet^{ΔΔ} mice have about 3.5-4.0% NK cells, whereas Eomes^{ΔΔ} have <1% of NK cells within lymphocytes. For Figure 6h, control and T-bet^{ΔΔ} mice have 0.6-0.8% KRLG1+CD27+ liver NK cells within lymphocytes, which correspond to about 10-25% of liver NK cells. Similarly, 6j shows IFN-γ+ liver NK cells at 0.6-1.2% positive for control and Tbet^{ΔΔ}, yielding roughly 10-25% positive liver NK cells. In both of those situations, Eomes^{ΔΔ} liver NK cells are very low to not detectable. In contrast, liver ILC-1 are overall less frequent (Figure 6i, k) with specific deficits in T-bet^{ΔΔ} setting only. This data presentation allows the reader to see that in the WT setting, the overall frequency of IFN-γ+ liver ILC-1s (6k) is lower compared to IFN-γ+ NK cells (6j). This data presentation has been clarified in the **Figure Legend**.

Additionally, in Figure 6b, T-bet KO mice appear to be somewhat protected from MCMV infection relative to WT mice, even if the difference is not statistically significant. The authors could speculate on potential mechanisms for this observation. For example, is there a specific NK/ILC1 population that is lost in T-bet KO mice that might contribute to immune pathology during infection? Addressing this could enhance the interpretation of the data.

Thank you for this insightful comment. While we agree that the survival data looks similar overall between WT and T-bet^{DD}, the T-bet^{DD} curve was higher (100% survival) but not statistically significant from the WT mice. The main population of NK/ILC1 lost in Tbet^{ΔΔ} are liver ILC-1, and while speculation, we have expanded the **Discussion** (paragraph 5-6) to point this out, and the larger literature surrounding immune pathology during a viral infection.

Minor points:

• Because the lung contains many vasculature-associated cells, is the reduction observed in total lung NK (and in Stage IV NK cells from lung) merely be a function of a reduction in blood NK cells? Perhaps IV labeling would help to determine whether tissue-resident cells are reduced as well.

We agree that lung NK cells are predominantly stage IV and prior reports have indicated they are vasculature associated. Respectfully, we felt that additional experiments to perform IV labeling to identify vascular-accessible

vs. tissue resident while of interest were beyond the scope of the current manuscript. Instead, we identify this possibility and propose this as a future direction in the **Discussion (Paragraph 3)**.

- In the discussion, could the authors expand speculation as to why Stage IV NK cells in the bone marrow are found at similar/higher levels in T-bet/Eomes dKO mice despite the opposite being found in all other tissues? Might Eomes play a role in promoting egress from the BM in the most mature NK cells?

We have expanded the **Discussion** (paragraph 3) related to bone marrow NK cells and their relative lack of a T-bet^{DD} phenotype in that tissue compartment.

- Is there a reason why the authors use tamoxifen chow in Fig. 6 as opposed to i.p. injections as performed in all previous experiments?

We use tamoxifen chow in Figure 6 to eliminate any impact of any confounding gavage bolus-associated impacts on proliferation of NK cells in this vivo model that included MCMV response. This approach has been used in other studies of Ncr1-iCreERT2 mice with similar results as bolus gavage (e.g., PMID: 40853248).

- Others: Figure 1g should be percent on the axis – Figure 5 frequency of lymphocytes

Thank you, we have reviewed all axes within the Figures and corrected as needed.

Reviewer #3 (Remarks to the Author):

In their manuscript, “Tissue-specific requirement for T-bet in NKp46+ innate lymphoid cell homeostasis, function, and response to MCMV,” Wong et al. use tamoxifen-inducible NKp46-Cre-ERT2 mice [PMID: 34521844] to investigate the roles of the T-box transcription factors T-bet and Eomes in mature NKp46+ innate lymphoid cells (NK cells, ILC1s, and NKp46+ ILC3s). Through conditional and inducible deletion of Tbx21 in fully developed NKp46+ cells, the authors uncover tissue- and subset-specific dependencies on the continuous expression of this transcription factor. Their findings demonstrate that sustained T-bet expression is critical for the survival of liver-resident ILC1s but dispensable for ILC1s in the small intestine, highlighting a tissue-specific role for T-bet in ILC1 homeostasis. In contrast, while T-bet deletion did not affect NK cells or ILC1s in the small intestine, it markedly compromised the survival of NKp46+ ILC3s, demonstrating subset-specific roles of T-bet in shaping NKp46+ ILC homeostasis. Furthermore, inducible deletion of T-bet modestly affected the survival of mature (stage IV) NK cells in the spleen and lung, leading to reduced anti-tumor immune responses in the lungs of mice injected with B16-F10 melanoma. Lastly, by contrasting the effects of Tbx21 and Eomes deletion in NKp46-expressing cells, the authors demonstrate that NK cells, rather than ILC1s, are essential for mounting a protective response against MCMV infection.

Major Concerns:

While the study highlights some new results, its overall impact is undermined by a lack of conceptual novelty, insufficient exploration of broader implications, and an overly descriptive presentation.

Thank you for this feedback and we agree that our synthesis with published data on T-bet and Eomes warranted expansion, which in turn highlights the conceptual advancements in this study. We respectfully disagree that new concepts are not advanced and rigorously supported by use of an Ncr1-specific, tamoxifen-inducible Cre model. The findings of loss-of-function of T-bet and T-bet/Eomes provide clear phenotypes and are applied in the broad context of viral infection. For example, use of this approach identified that Eomes+ NK cells, but not T-bet+ ILC1s are required for host protection against MCMV, contrary to prior findings using other model systems. Here are some of the major findings that are advanced using this model, which also set the stage for new avenues of study:

- Induced loss of T-bet within Ncr1+ cells results in a modest NK cell deficiency in the blood, lung, but not in the lymph node or bone marrow
- Induced loss of T-bet within Ncr1+ cells markedly reduces CD27-CD11b+ Stage IV NK cells
- The loss of Stage IV NK cells in Tbet$^{\Delta\Delta}$ mice is associated with increased apoptosis
- Stage II NK cells that are Tbet$^{\Delta\Delta}$ have reduced cytokine-induced IFN-γ production
- Stage III and IV NK cells that are Tbet$^{\Delta\Delta}$ have reduced degranulation in response to YAC-1 targets, associated with a modest reduction in in vitro cytotoxicity
- The NK cell alterations within Ncr1-specific Tbet$^{\Delta\Delta}$ mice result in reduced rejection of B2m-/- splenocytes and reduced in vivo tumor metastases control
- Induced loss of Tbet results in the rapid loss of liver ILC1s, and increased apoptosis, identifying the need for sustained Tbet transcriptional programs for this ILC
- Analysis of the SI LP revealed that NKp46+ ILC3 require T-bet for survival over one week, whereas SI LP ILC1s were not affected over this time frame
- The concurrent deletion of Tbet and Eomes within mature Ncr1+ cells results in the profound reduction in NK cells throughout the mouse
- Stage IV NK cells in the bone marrow were resistant to the impacts of Tbet and Tbet/Eomes deletion, suggesting alternative homeostatic transcriptional programs
- Mice with conditional, inducible deletion of T-bet lost ILC1s yet controlled MCMV infection
- Mice with conditional, inducible deletion of Eomes lost NK cells and succumbed to fatal MCMV infection

The use of an inducible conditional knockout model allows for precise dissection of T-bet and Eomes functions in fully developed NK cells and ILCs, overcoming limitations of previous germline knockout or constitutive NKp46-Cre+ conditional knockout mice. Additionally, the authors provide a phenotypic analysis of the transcriptional and functional consequences of T-bet loss in NKp46+ ILCs across multiple tissues. These aspects provide a strong foundation for mechanistically dissecting tissue-specific dependencies of T-box transcription factors in innate lymphoid cells.

We thank the reviewer for appreciating the technical innovation, strengths, and importance of NKp46-conditional, inducible T-box transcription factor mouse models. We agree that using these innovative approaches provides a clear picture of their impact on NK/ILC biology and is needed to avoid confounding effects within alternative models.

Despite these strengths, the manuscript struggles to advance the field conceptually. Many of the findings reiterate known roles of T-bet and Eomes in NK cells, such as their contributions to NK cell maturation and cytotoxicity [PMID: 34521844, PMID: 29440505, PMID: 24516120]. Although the tissue-specific differences observed in this study are interesting, they represent incremental advances rather than groundbreaking insights. For instance, the observation that liver ILC1s require sustained T-bet expression while small intestine ILC1s do not is intriguing but insufficiently explored within the broader framework of immune regulation. The discussion section is another area where the manuscript falls short. It is wordy, unfocused, and often reiterates results rather than synthesizing them into a coherent narrative. Overall, while the technical execution is commendable, the manuscript falls short of conceptual depth. The study would benefit from providing mechanistic insights and presenting a more cohesive narrative to highlight its contribution to the field.

Thanks for the comment, and the opportunity to refocus aspects of the Discussion on prior T-bet findings in the field. We certainly appreciate that this is not the first study of T-bet and Eomes in NK cells, nor the first to implicate their role in specific mature NK cell functions. We discuss the limitations of prior models, and highlight the importance of using these conditional, inducible genetic models rather than global knockout approaches, global inducible models, or even conditional (constitutive) models, to best understand their role in matured NK cells. In terms of conceptual impact, by developing these models, we defined the relative importance of NK cells and ILC1s to protect against lethal viral infection with minimal manipulation of the mice. Prior work using alternative models implicated ILC1s as the critical group 1 ILC required for host protection against MCMV (PMID: 29056343). Using genetic models that clearly eliminate ILC1 (T-bet) vs. NK cells (Eomes) with temporal control after NK cell development and minimal confounding factors, yields very different results. In our view, this is a critical finding in the NK/ILC1 field, and a major conceptual contribution. Additional conceptual advances are listed above.

To specifically address the novel findings in our study, and better place them in the context of the papers referenced by the Reviewer, we have re-written much of the Discussion and edited the Introduction, including more extensive discussion of the papers called out by the reviewer.
